# Supplementary material for: The biosynthesis, degradation, and function of cell wall β‐xylosylated xyloglucan mirrors that of arabinoxyloglucan
Source: New Phytol. 2023 Oct 12;240(6):2353–71. doi: 10.1111/nph.19305 (PMC10952531; doi:10.1111/nph.19305)
Supplement: Supplementary file 3 — Fig. S1 Unrooted maximum‐likelihood phylogeny of GT47‐A sequences from a range of asterid and nonasterid species, showing recent expansion of the GT47‐AIII clade in asterids. Fig. S2 GT47‐AIII subtree from Figs 2 and S1, fully labelled. Fig. S3 Reported confidence metrics for the AlphaFold model of AtXLT2. Fig. S4 Individual GT47‐A T1 transformants in mur3‐3 background. Fig. S5 Individual GT47‐A T1 transformants in xlt2 mur3‐1 background. Fig. S6 No‐enzyme controls for endo‐XGase digestions of transgenic Arabidopsis material. Fig. S7 SDS‐PAGE gels of purified BoGH43A and BoGH43B. Fig. S8 Initial time courses of BoGH43A and BoGH43B activity on para‐nitrophenyl (pNP) glycosides pNP‐α‐Araf, pNP‐β‐Xyl, and pNP‐α‐Arap in order to screen for potential previously unreported activities. Fig. S9 pH optima and Michaelis–Menten curves for BoGH43A and BoGH43B activity on para‐nitrophenyl (pNP) glycosides. Fig. S10 No‐enzyme controls for endo‐XGase digestions of plants exhibiting XXXG‐type xyloglucan. Fig. S11 MALDI‐TOF mass spectrometry analysis of endo‐xyloglucanase products from olive leaf, argan leaf, and blueberry fruit skin. Fig. S12 Sensitivity of olive, argan, and blueberry endo‐XGase products to α1,2/3‐arabinofuranosidase and β1,2‐xylosidase. Fig. S13 Spectrum from tandem mass spectrometry (MS–MS) with collision‐induced dissociation (CID) of uncharacterised blueberry xyloglucan oligosaccharide. Fig. S14 Sequential digestion of an unidentified endo‐XGase product from blueberry skin xyloglucan. Fig. S15 Partial characterisation of endo‐XGase products from Coffea arabica ‘Catimor’ leaf xyloglucan. Fig. S16 No‐enzyme controls for endo‐XGase digestions of plants exhibiting XXGG‐/XXGn‐type xyloglucan. Fig. S17 Sensitivity of endo‐XGase products from Catharanthus roseus leaf xyloglucan to various exo‐glycosidases. Fig. S18 Sensitivity of endo‐XGase products from Coffea arabica ‘Catimor’ root xyloglucan to various exo‐glycosidases. Fig. S19 Sensitivity of endo‐XGase products fr [file NPH-240-2353-s002.pdf]

New Phytologist **Supporting Information**

Article title: The biosynthesis, degradation, and function of β-xylosylated xyloglucan mirrors that of arabinoxyloglucan

Authors: Louis F. L. Wilson, Stefanie Neun, Li Yu, Theodora Tryfona, Katherine Stott, Florian Hollfelder, Paul Dupree

Article acceptance date: 02 September 2023

The following Supporting Information is available for this article:

Dataset S1 Alignment of GT47-A sequences from a range of asterid and non-asterid species, truncated to GT47 domain and used to produce main tree. FASTA format (may be opened with any text editor [Notepad etc.], phylogenetic software [Mega etc.], or online alignment viewer [MView, ESPript etc.]).

Dataset S2 Unrooted maximum-likelihood phylogeny of GT47-A sequences from a range of asterid and non-asterid species; raw tree. Newick format (may be opened with any graphical viewer for phylogenetic trees [FigTree, Mega etc.]).

Fig. S1 Unrooted maximum-likelihood phylogeny of GT47-A sequences from a range of asterid and non-asterid species, showing recent expansion of the GT47-AIII clade in asterids.

Fig. S2 GT47-A_{III} subtree from Fig. 2 and Fig. S1, fully labelled.

Fig. S3 Reported confidence metrics for the AlphaFold model of *At*XLT2.

Fig. S4 Individual GT47-A T_1 transformants in *mur3-3* background.

Fig. S5 Individual GT47-A T_1 transformants in *xlt2 mur3-1* background.

Fig. S6 No-enzyme controls for *endo*-XGase digestions of transgenic Arabidopsis material.

Fig. S7 SDS-PAGE gels of purified *Bo*GH43A and *Bo*GH43B.

Fig. S8 Initial timecourses of *Bo*GH43A and *Bo*GH43B activity on *para*-nitrophenyl (*p*NP) glycosides *p*NP-α-Ara*f*, *p*NP-β-Xyl, and *p*NP-α-Ara*p* in order to screen for potential previously

unreported activities.

Fig. S9 pH optima and Michaelis-Menten curves for *Bo*GH43A and *Bo*GH43B activity on *para*-nitrophenyl (*p*NP) glycosides.

Fig. S10 No-enzyme controls for *endo*-XGase digestions of plants exhibiting XXXG-type xyloglucan.

Fig. S11 MALDI-TOF mass spectrometry analysis of *endo*-xyloglucanase products from olive leaf, argan leaf, and blueberry fruit skin.

Fig. S12 Sensitivity of olive, argan, and blueberry *endo*-XGase products to α1,2/3-arabinofuranosidase and β1,2-xylosidase.

Fig. S13 Spectrum from tandem mass spectrometry (MS–MS) with collision-induced dissociation (CID) of uncharacterised blueberry xyloglucan oligosaccharide.

Fig. S14 Sequential digestion of an unidentified *endo*-XGase product from blueberry skin xyloglucan.

Fig. S15 Partial characterisation of *endo*-XGase products from *Coffea arabica* 'Catimor' leaf xyloglucan.

Fig. S16 No-enzyme controls for *endo*-XGase digestions of plants exhibiting XXGG-/XXG_n-type xyloglucan.

Fig. S17 Sensitivity of *endo*-XGase products from *Catharanthus roseus* leaf xyloglucan to various *exo*-glycosidases.

Fig. S18 Sensitivity of *endo*-XGase products from *C. arabica* 'Catimor' root xyloglucan to various *exo*-glycosidases.

Fig. S19 Sensitivity of *endo*-XGase products from *C. arabica* 'Catuai Amarelo' root xyloglucan to

various *exo*-glycosidases.

Fig. S20 Sensitivity of *Coffea arabica* ‘Catuai Amarelo’ root *endo*-XGase products to α1,2/3-arabinofuranosidase and β1,2-xylosidase.

Fig. S21 Characterisation of *endo*-XGase products from kiwi fruit skin xyloglucan.

Table S1 Genomic data sources.

Table S2 De novo-synthesised coding sequences for Golden Gate assembly.

Table S3 PCR primers used to amplify DNA parts for Golden Gate assembly.

Table S4 *Exo*-glycosidases used in this work.

Table S5 ^{1}H and ^{13}C NMR assignments for XUXG oligosaccharide.

Table S6 Kinetic parameters for *Bo*GH43A/B activity on *para*-nitrophenyl glycosides.

Methods S1 Glycosyl hydrolase expression and purification.

Methods S2 Oligosaccharide purification.

Fig. S1 Unrooted maximum-likelihood phylogeny of GT47-A sequences from a range of asterid and non-asterid species, showing recent expansion of the GT47-A$_{III}$ clade in asterids. See Fig. S2 or Table S1 for a full list of species. Known donor substrate specificities are annotated with sugar symbols (as designated in the key). Sequences were truncated to the GT47 domain prior to phylogenetic inference using IQ-TREE. Branch lengths indicate average number of subsititutions per site (refer to scale bar). Support values at important splits represent percentage replication within 1,000 ultra-fast bootstrap pseudo-replicates. GT47-A subclades are annotated with roman numerals (I–VII) as in Yu *et al*. (2022). GT47-A subclade III (GT47-A$_{III}$) is highlighed in grey; lower level subclades within GT47-A$_{III}$ (*a*, *b*, *c* etc.) are also annotated.

Since *Sl*MUR3 is annotated as two separate loci in Phytozome (Solyc09g064470.3.1 and Solyc09g064480.1.1), its sequence was automatically was filtered out by the length threshold imposed during quality control. However, our previous phylogeny (Yu *et al.*, 2022; DOI: 10.1093/plcell/koac238) grouped it in GT47-A$_{VI}$.

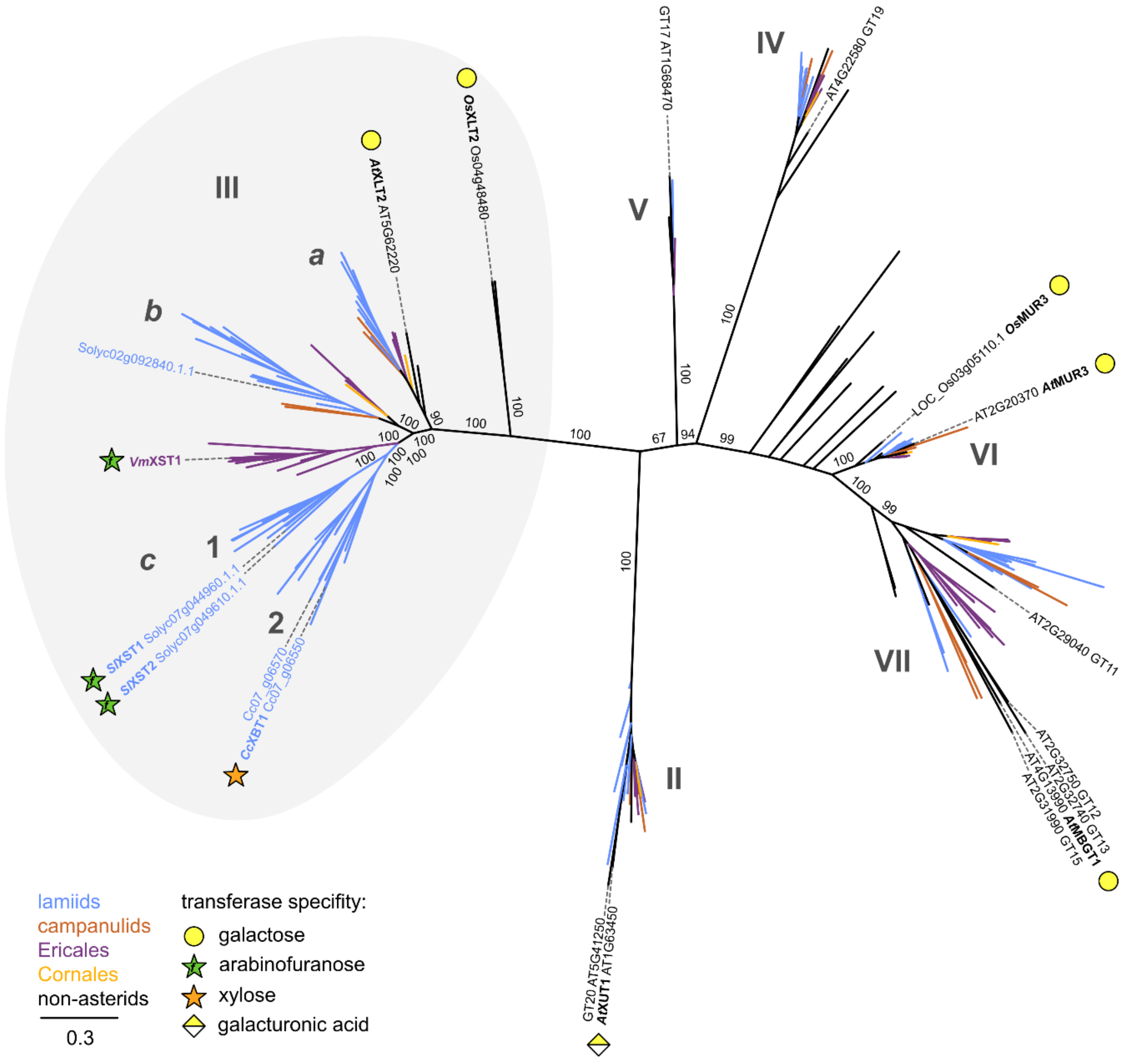

Fig. S2 GT47-A$_{III}$ subtree from Fig. 2 and Fig. S1, fully labelled. Horizontal branch lengths indicate average number of subsititutions per site (refer to scale bar). Support values at each split represent percentage replication within 1,000 ultra-fast bootstrap pseudo-replicates. See Table S1 for full information on the source of the sequences.

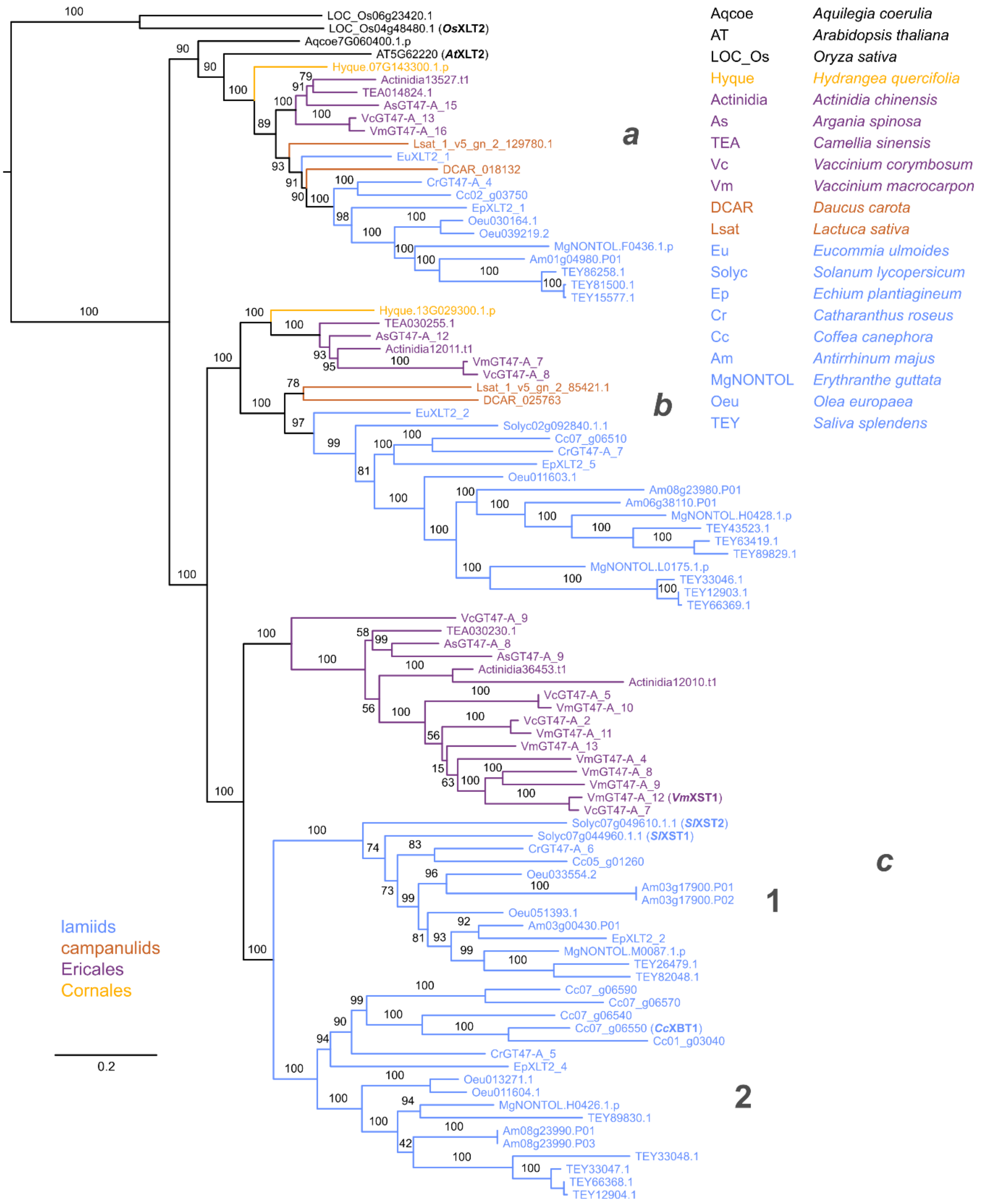

Fig. S3 Reported confidence metrics for the AlphaFold model of *At*XLT2. Figures were generated at the AlphaFold Protein Structure database (https://alphafold.ebi.ac.uk). **a** Per-residue confidence scores representing the predicted local distance difference test (pLDDT) for full-length *At*XLT2, including transmembrane helix, stem domain, and catalytic domain. Residues with pLDDT < 50 are predicted to be disordered. **b** Predicted aligned error plot.

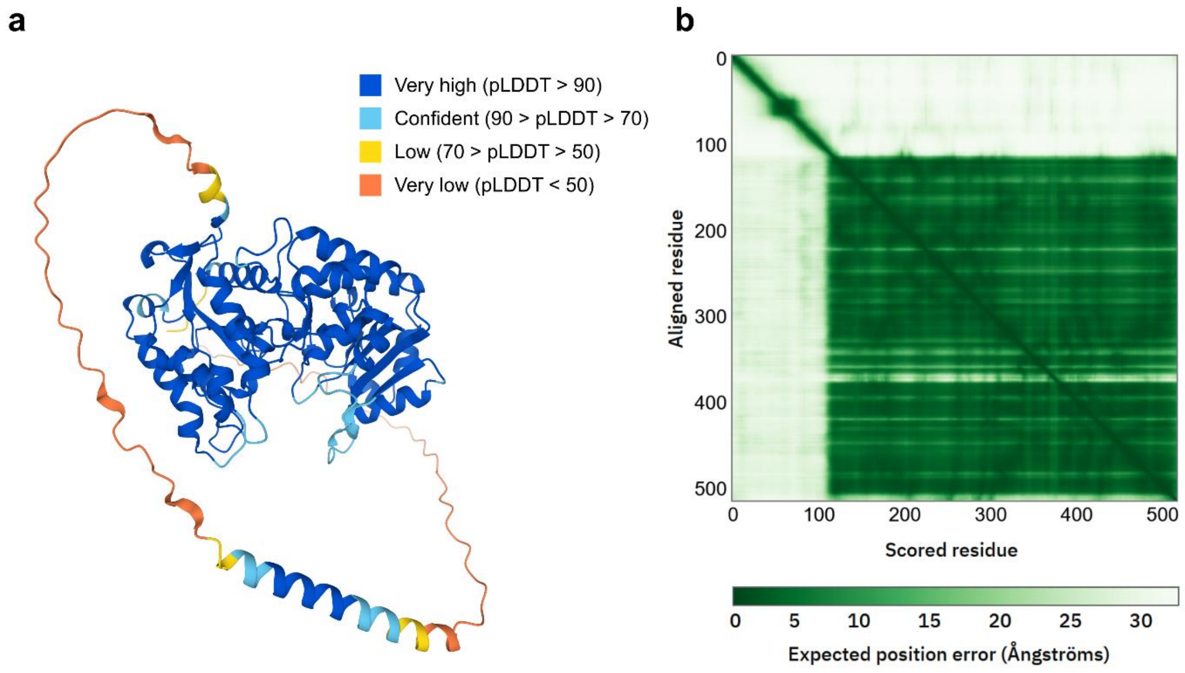

Fig. S4 Individual GT47-A T_1 transformants in Arabidopsis *mur3-3* background. Plants were photographed after six weeks of growth. For the transgenic plants, each plant is a separate transgenic line (lines are labelled #1, #2, etc.). Transgenes were expressed under the promoter of xyloglucan α-xylosyltransferase XXT2. Expression of *Cc*XBT1 resulted in full rescue of *mur3-3*'s stunted growth, whereas expression of *Vm*XST1 afforded only partial complementation. Transformation with Cc07_g06570 had no effect apparent on the phenotype of *mur3-3*.

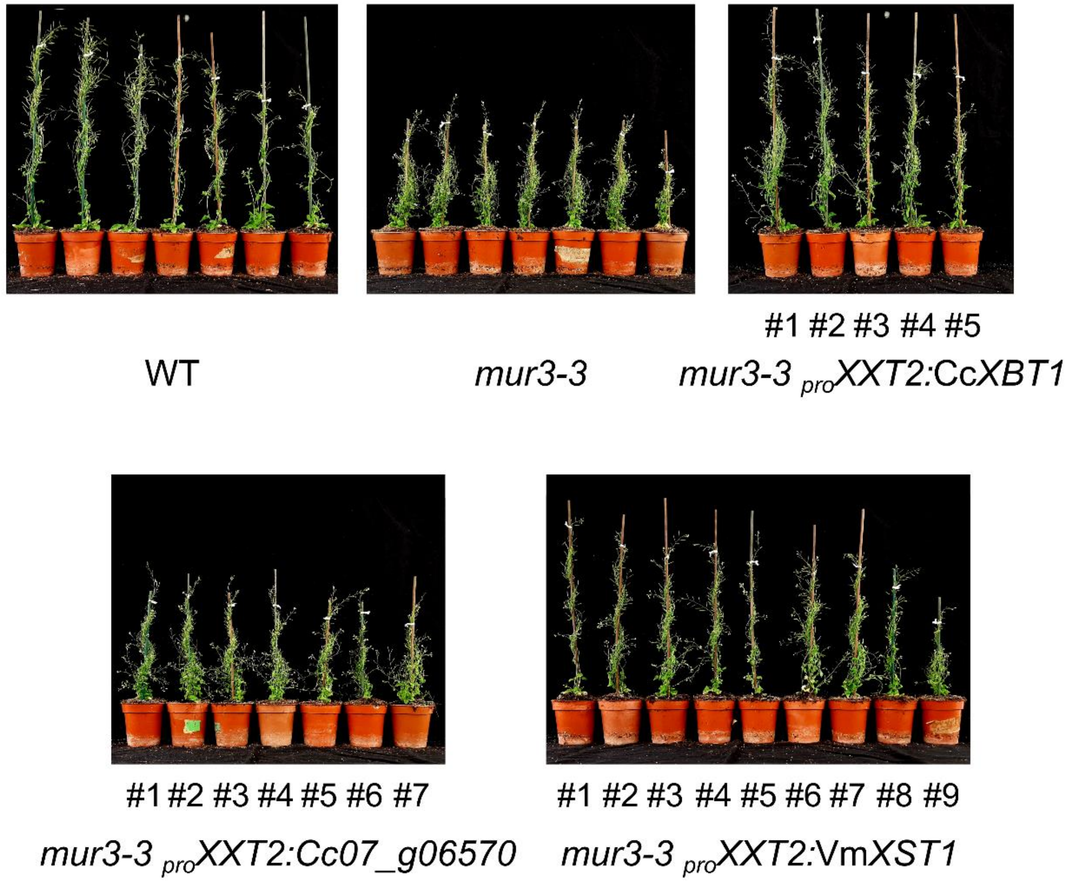

Fig. S5 Individual GT47-A T_1 transformants in Arabidopsis *xlt2 mur3-1* background. a Plant growth phenotypes. Plants were photographed after six weeks of growth. For the transgenic plants, each plant is a separate transgenic line. **b** PACE analysis of *endo*-xyloglucanase (*endo*-XGase) products from transgenic cell wall material. Hemicellulose was extracted by alkali treatment of leaf alcohol insoluble residue (AIR) and digested with *Aa*XEG *endo*-XGase. Products were subsequently derivatised with a fluorophore and separated by electrophoresis. Individual transgenic lines are numbered in accordance with the photographs in panel **a**.

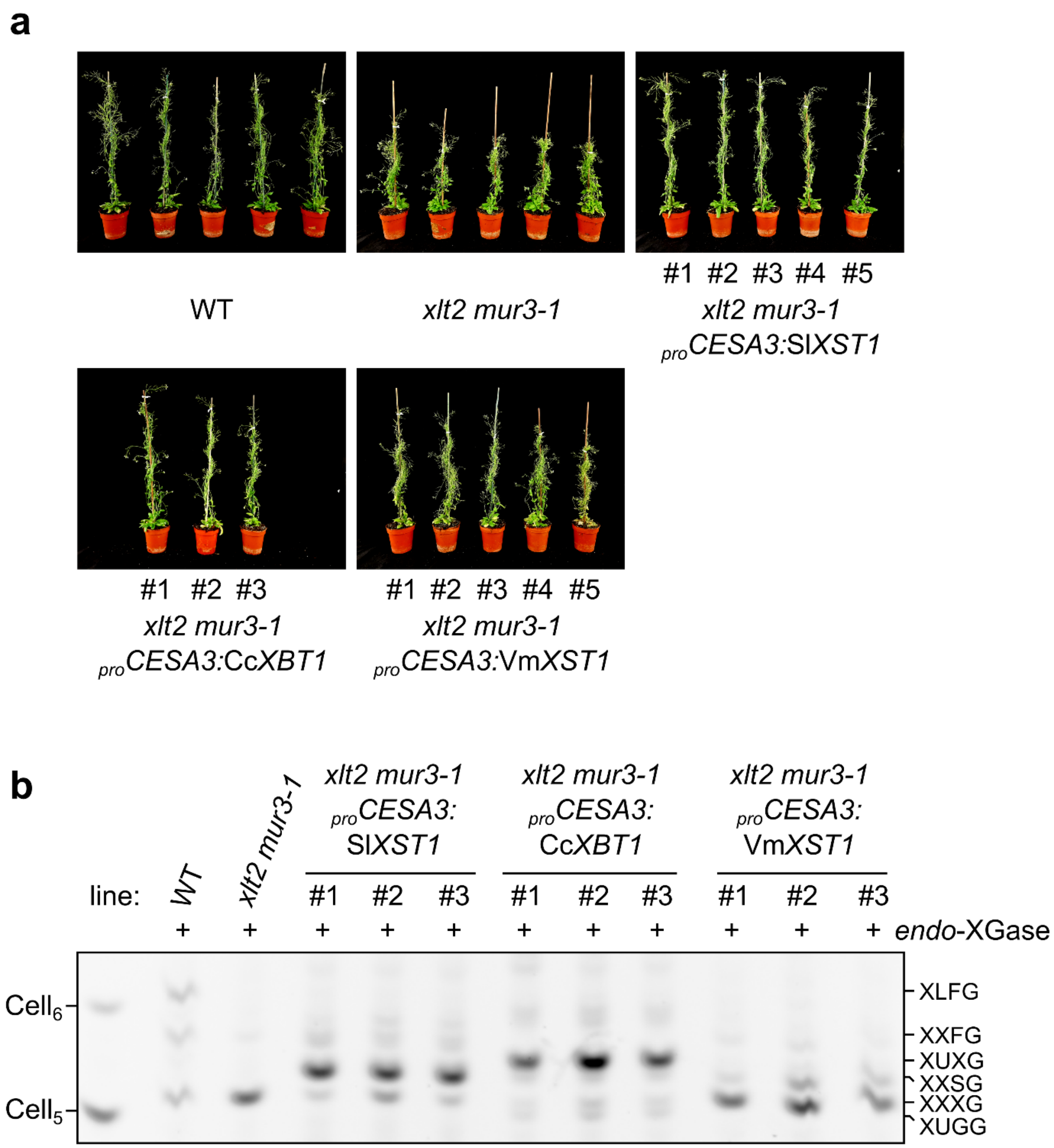

Fig. S6 No-enzyme controls for *endo*-XGase digestions of transgenic Arabidopsis material. Alkali-extracted hemicellulose from transgenic plants was treated with or without *Aa*XEG *endo*-XGase to confirm that the bands displayed in Fig. 3 result from specific hydrolysis by *endo*-XGase, as opposed to background contamination. Products were subsequently derivatised with a fluorophore and separated by electrophoresis.

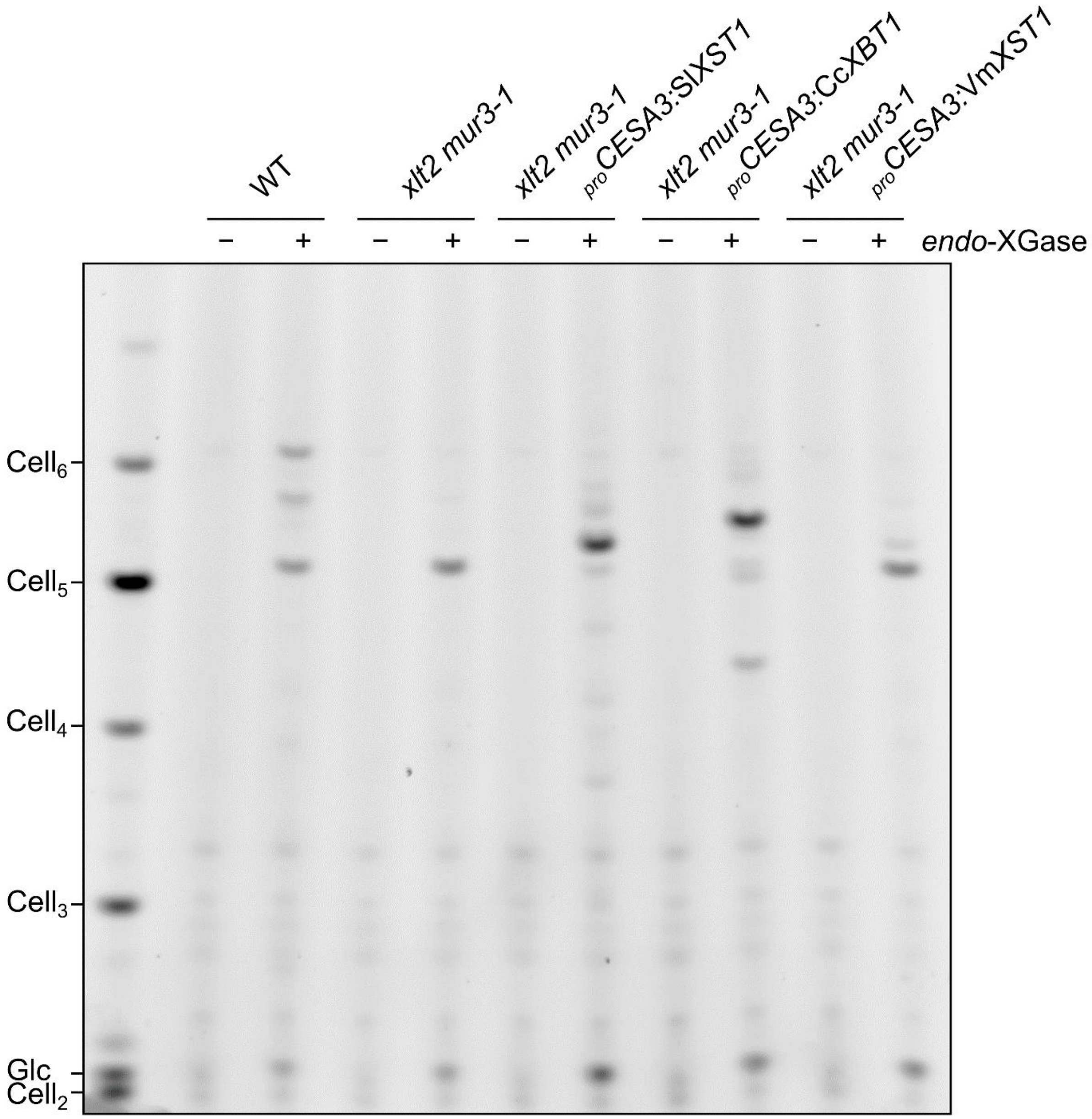

Fig. S7 SDS-PAGE gels of purified *Bo*GH43A and *Bo*GH43B. Glycosyl hydrolases *Bo*GH43A and *Bo*GH43B from *Bacteroides ovatus* ATCC 8483 were expressed in and purified from *E. coli*. Purified proteins migrated consistently with their theoretical molecular weight (*Bo*GH43A: 57.6 kDa; *Bo*GH43B: 57.2 kDa).

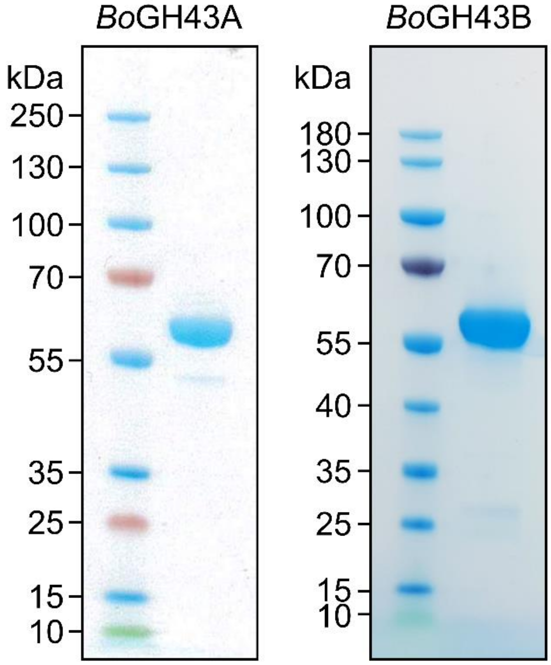

Fig. S8 Initial timecourses of *Bo*GH43A and *Bo*GH43B activity on *para*-nitrophenyl (*p*NP) glycosides *p*NP-α-Ara*f*, *p*NP-β-Xyl, and *p*NP-α-Ara*p* in order to screen for potential previously unreported activities. Purified glycosyl hydrolases *Bo*GH43A and *Bo*GH43B from *Bacteroides ovatus* ATCC 8483 were assayed using synthetic substrates. *p*NP glycosides were incubated at 1 mM concentration with 2 µM enzyme in 50 mM buffer, pH 7.0, T = 25°C. Reaction progress was monitored by observing A_{405} at 1 min intervals. Circles represent individual data points. The lack of hydrolysis of *p*NP-α-Ara*p* even at 1 mM substrate concentration indicates a lack of activity and therefore the kinetics were not investigated further.

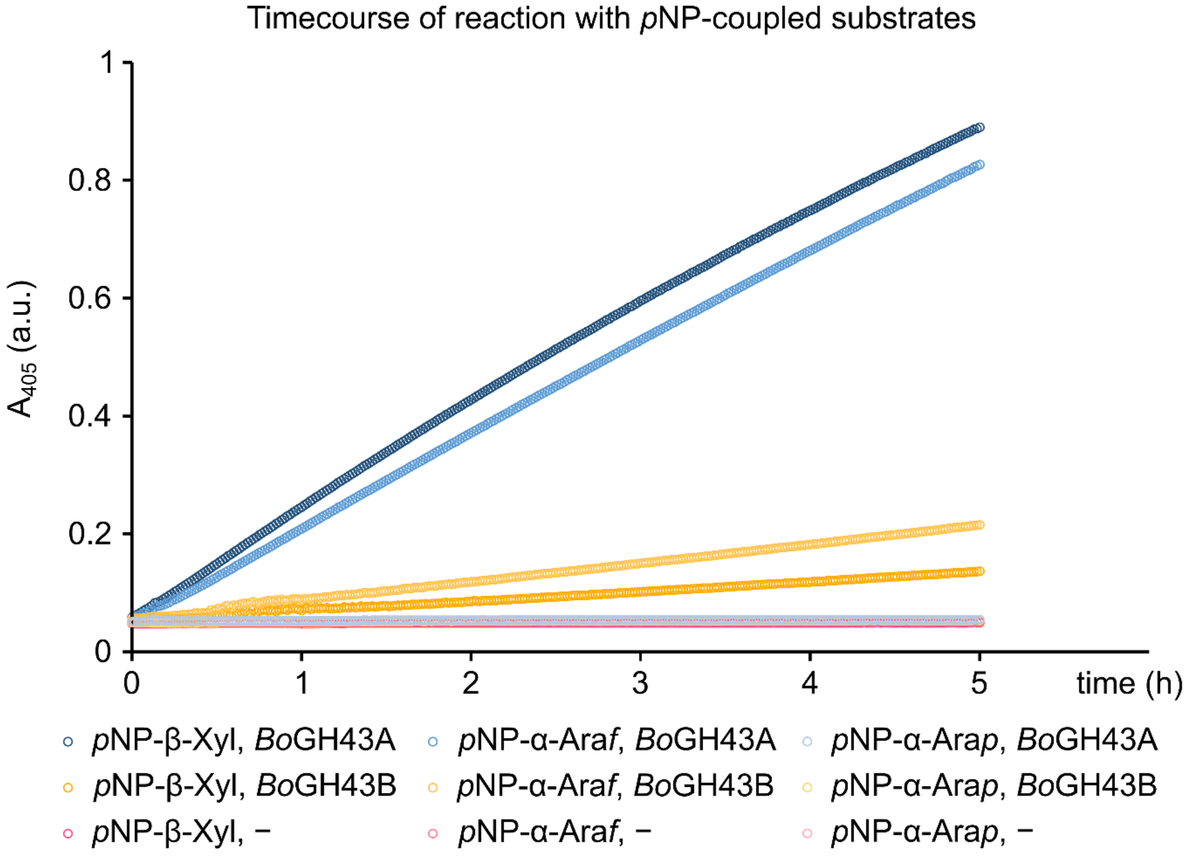

Fig. S9 pH optima and Michaelis-Menten curves for *Bo*GH43A and *Bo*GH43B activity on *para*-nitrophenyl (*p*NP) glycosides. Purified glycosyl hydrolases *Bo*GH43A and *Bo*GH43B from *Bacteroides ovatus* ATCC 8483 were assayed using synthetic substrates. **a,b** Determination of pH optima. Initial reaction velocities of **a** *Bo*GH43A on *p*NP-β-Xyl and **b** *Bo*GH43B on *p*NP-α-Ara*f*, determined by monitoring A_{405} with 1 mM substrate and 2 µM enzyme in 50 mM buffer. Note that neither enzyme has any activity in Tris-HCl buffer. Tris has been observed to bind strongly to the active site of *Bo*GH43A in crystal structures (Hemsworth *et al.*, 2016). **c–f** Kinetic curves for *Bo*GH43A and *Bo*GH43B activity on *p*NP-α-Ara*f* (**c,d**) and *p*NP-β-Xyl (**e,f**). All kinetic measurements were carried out with 1 µM enzyme in 50 mM HEPES pH 7.5 at 20 °C. Reactions were monitored with A_{405} in a plate reader. Kinetic data are summarized in Table S6.

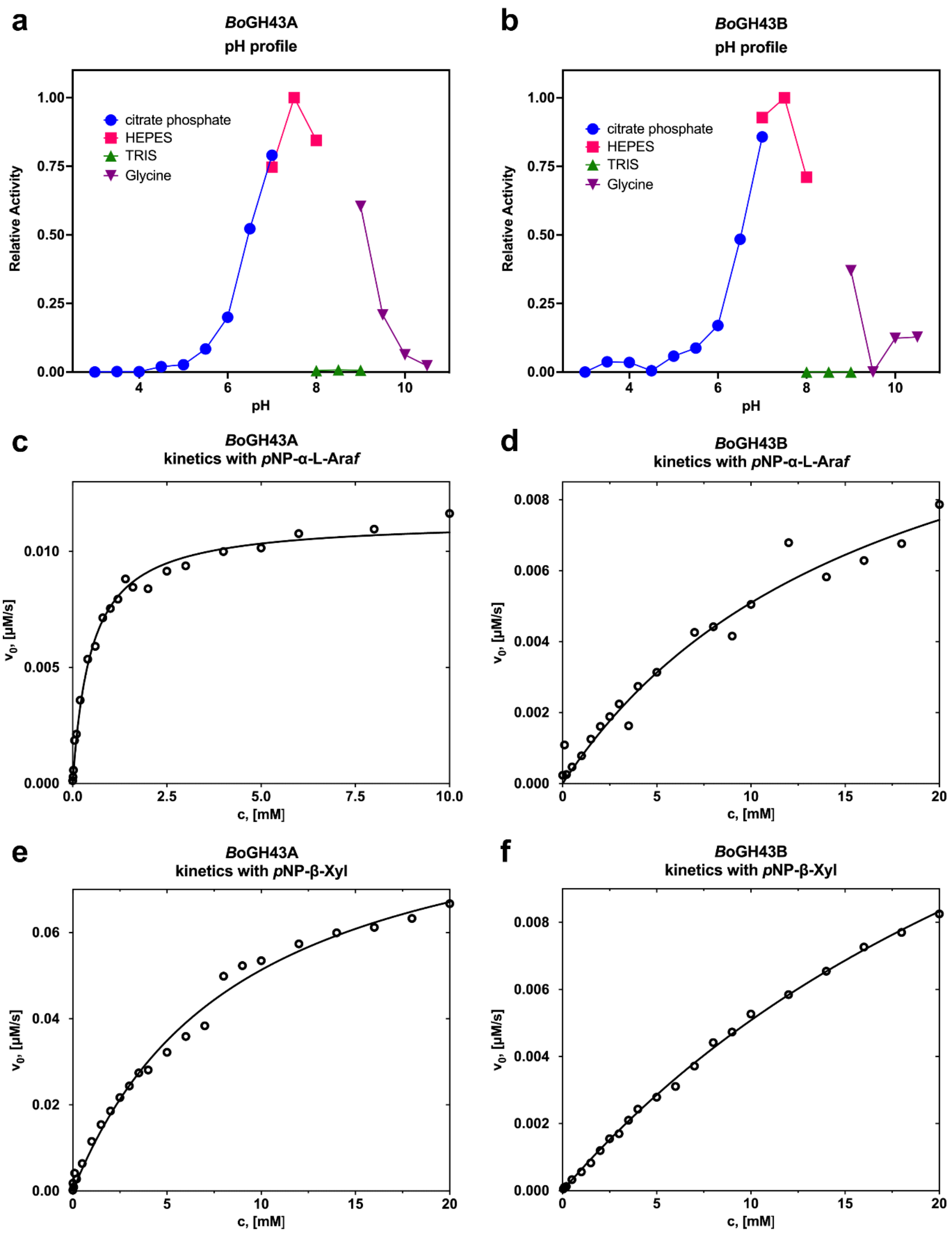

a
BoGH43A
pH profile
Relative Activity
citrate phosphate
HEPES
TRIS
Glycine
pH
b
BoGH43B
pH profile
c
BoGH43A
kinetics with pNP-α-L-Araf
v0, [µM/s]
c, [mM]
d
BoGH43B
kinetics with pNP-α-L-Araf
e
BoGH43A
kinetics with pNP-β-Xyl
f
BoGH43B
kinetics with pNP-β-Xyl

Fig. S10 No-enzyme controls for *endo*-XGase digestions of plants exhibiting XXXG-type xyloglucan. Alkali-extracted hemicellulose from transgenic plants was treated with or without *Aa*XEG endo-XGase to confirm that the bands displayed in Fig. 6 result from specific hydrolysis by *endo*-XGase, as opposed to background contamination. Products were subsequently derivatised with a fluorophore and separated by electrophoresis.

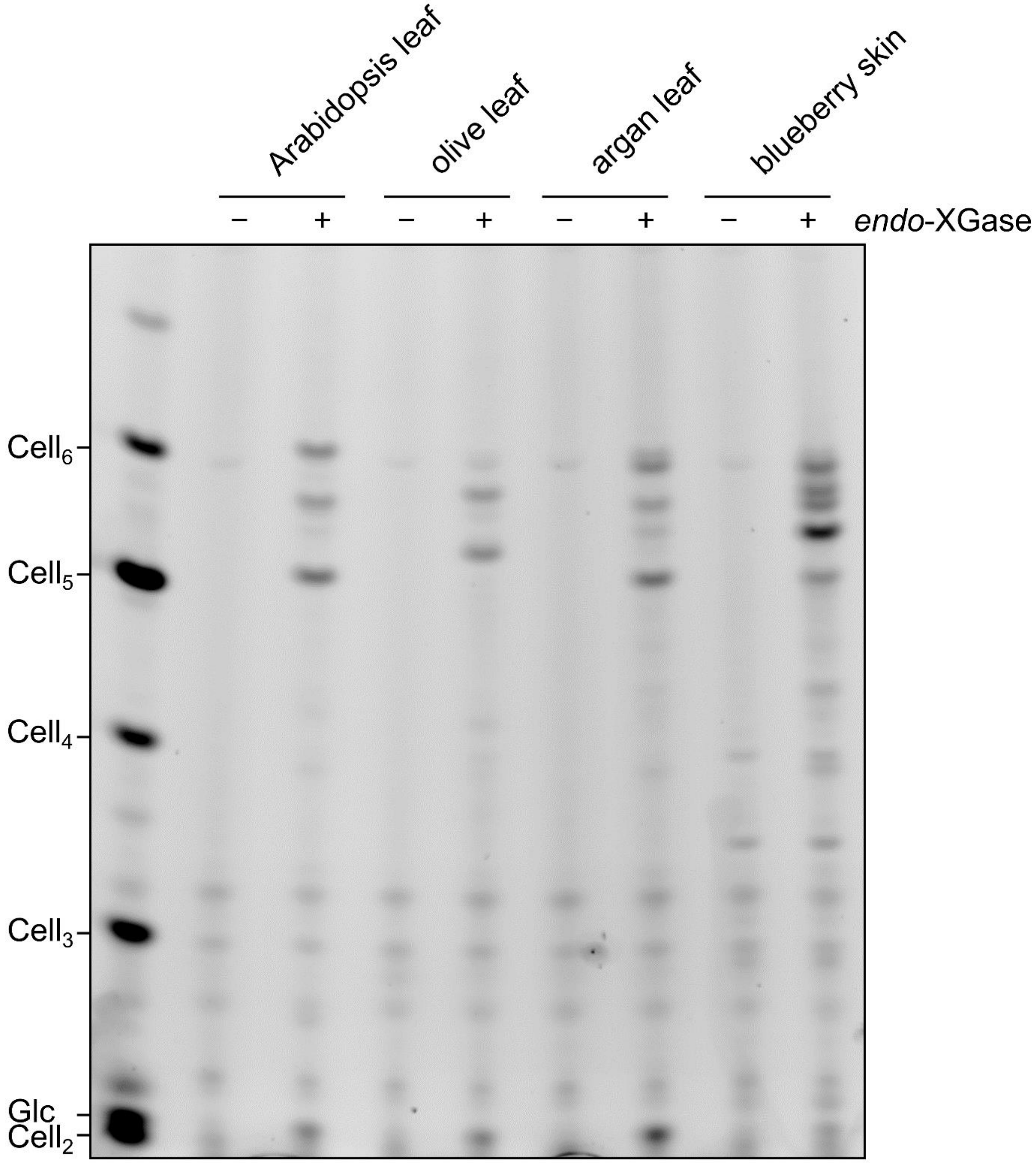

Fig. S11 MALDI-TOF mass spectrometry analysis of *endo*-xyloglucanase products from olive leaf, argan leaf, and blueberry fruit skin. Alkali-extracted hemicellulose was treated with *Aa*XEG *endo*-XGase. H = unknown hexose; P = unknown pentose.

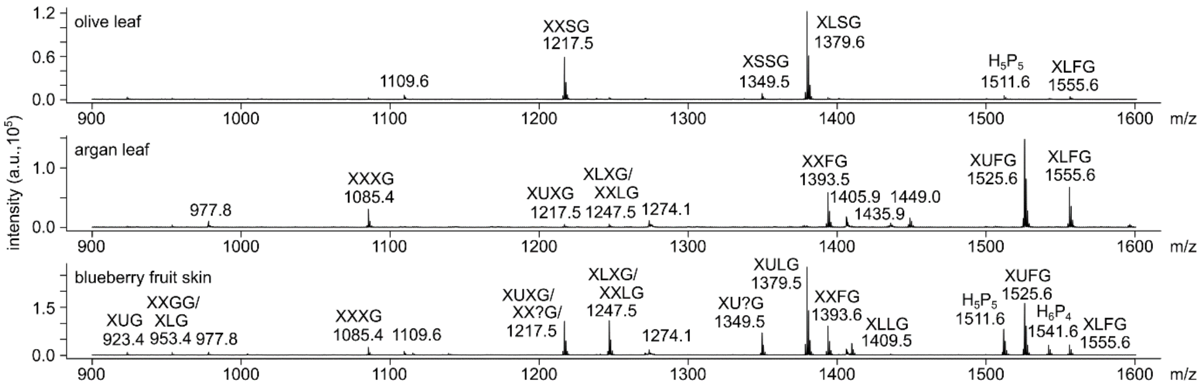

Fig. S12 Sensitivity of olive, argan, and blueberry *endo*-XGase products to α1,2/3-arabinofuranosidase and β1,2-xylosidase. Alkali-extracted hemicellulose from green tomato fruit or kiwi fruit skin was digested with *Aa*XEG *endo*-XGase before being subjected to ethanol precipitation. Oligosaccharides were then treated to combinatorial digestion with *Bb*AfcA α1,2-fucosidase (α1,2-Fuc-ase), Fam35 β-galactosidase (β-Gal-ase), *Cj*Abf51 α1,2/3-arabinofuranosidase (α1,2/3-Ara*f*-ase), and/or *Cg*GH3 β1,2-xylosidase. Products were subsequently derivatised with a fluorophore and separated by electrophoresis. **a** Digestion of *endo*-XGase from olive leaf. **b** Digestion of *endo*-XGase from argan leaf. **c** Digestion of *endo*-XGase from blueberry fruit skin.

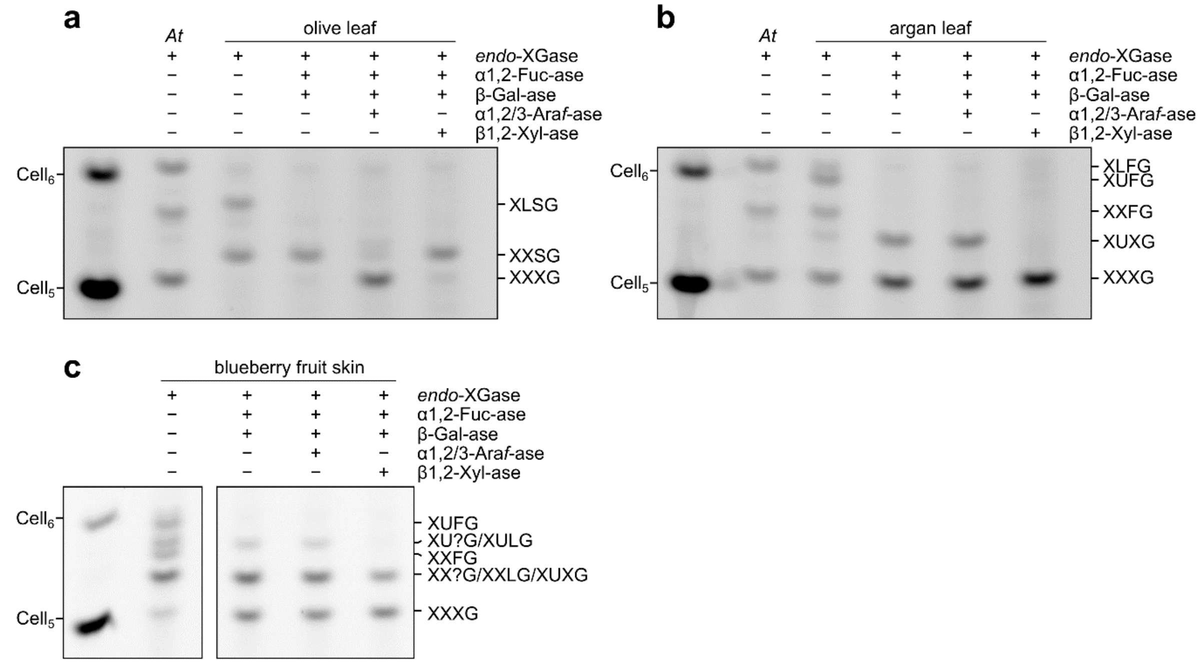

Fig. S13 Spectrum from tandem mass spectrometry (MS–MS) with collision-induced dissociation (CID) of uncharacterised blueberry xyloglucan oligosaccharide. The unidentified octasaccharide was semi-purified from alkali-treated blueberry skin AIR by treatment of *Aa*XEG *endo*-XGase products with *Bb*AfcA α1,2-fucosidase, Fam35 β-galactosidase, and *Bo*GH43A followed by size exclusion chromatography and reducing-end derivatisation with 2-aminobenzamide.

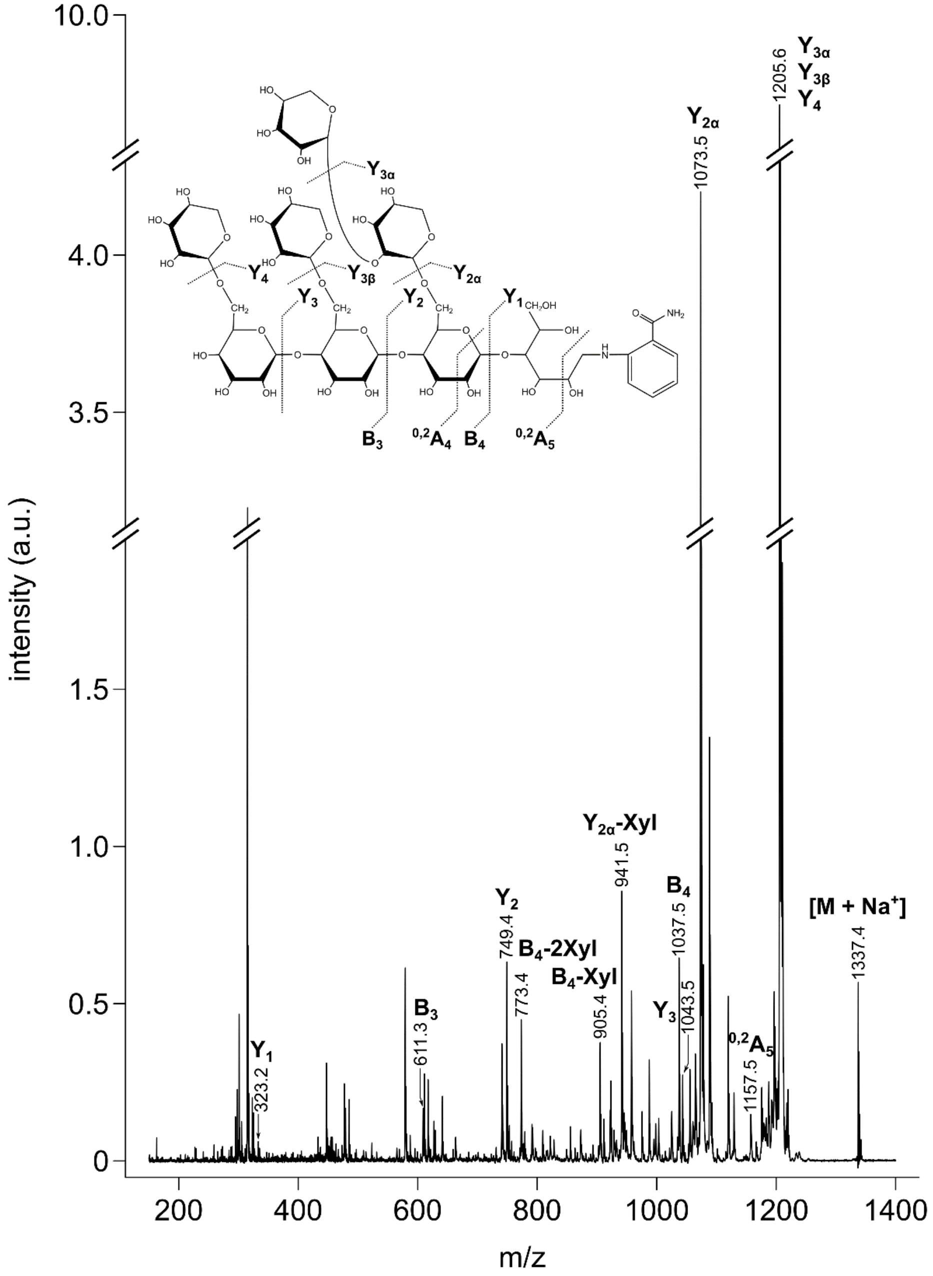

Fig. S14 Sequential digestion of an unidentified *endo*-XGase product from blueberry skin xyloglucan. Alkali-extracted hemicellulose from blueberry fruit skin was digested with *Aa*XEG *endo*-XGase before being subjected to ethanol precipitation. A sequential digest was then performed with *Bb*AfcA α1,2-fucosidase (α1,2-Fuc-ase), Fam35 β-galactosidase (β-Gal-ase), *Bo*GH43A, *E. coli* YicI non-reducing-end-specific xyloglucan α1,6-xylosidase (α1,6-Xyl-ase), and *An*GH3 β1,4-glucosidase (β1,4-Glc-ase). After simultaneous treatment with the first three enzymes, and separately after β1,4-Glc-ase digestion, products were isolated using a centrifugal filter (represented by dotted line). For α1,6-Xyl-ase digestions, ethanol precipitation (dotted line) was used to deactivate/remove enzyme.

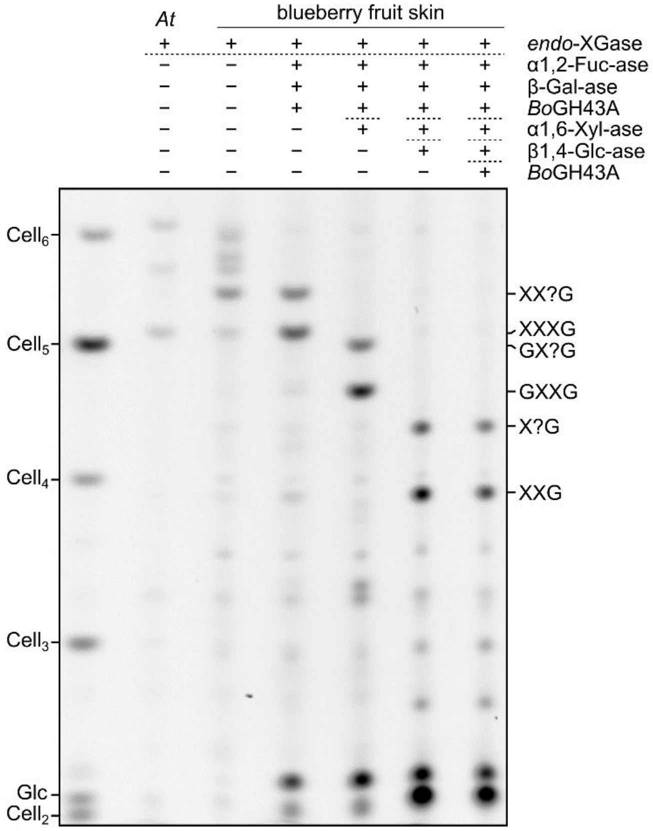

Fig. S15 Partial characterisation of *endo*-XGase products from *Coffea arabica* 'Catimor' leaf xyloglucan. Alkali-extracted hemicellulose from *C. arabica* 'Catimor' leaf was digested with *Aa*XEG *endo*-XGase before being subjected to ethanol precipitation. **a** Left: PACE gel comparing *endo*-XGase products released from xyloglucan of Arabidopsis, tomato, and *C. arabica*. Right: Combinatorial digest of *C. arabica endo*-XGase products with *Bb*AfcA α1,2-fucosidase (α1,2-Fuc-ase), Fam35 β-galactosidase (β-Gal-ase), and/or *Bo*GH43A. **b** MALDI-TOF mass spectrometry analysis of *C. arabica endo*-XGase products. H = unknown hexose; P = unknown pentose.

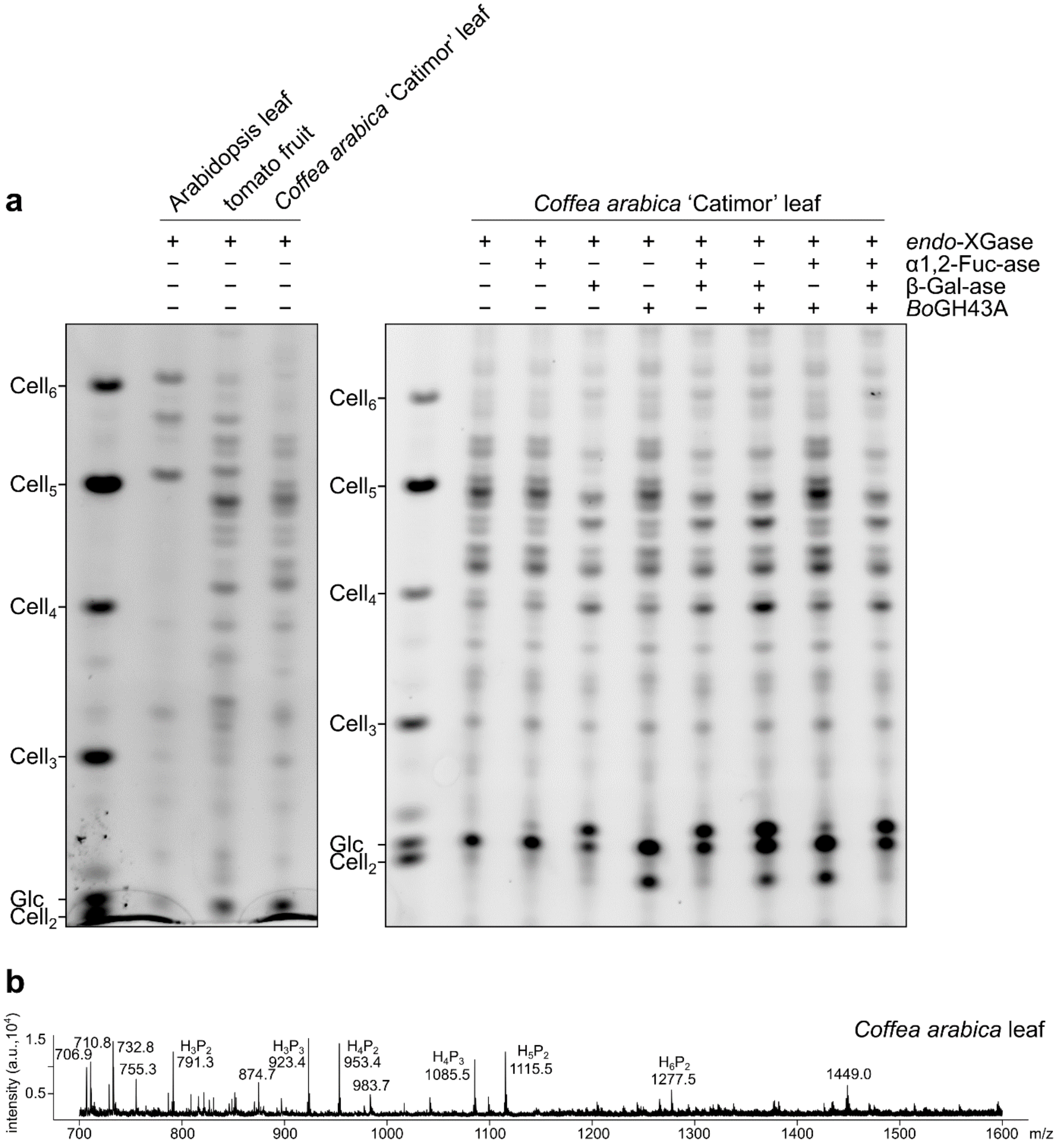

Fig. S16 No-enzyme controls for *endo*-XGase digestions of plants exhibiting XXGG-/XXG$_n$-type xyloglucan. Alkali-extracted hemicellulose from transgenic plants was treated with or without *Aa*XEG endo-XGase to confirm that the bands displayed in Fig. 7 result from specific hydrolysis by *endo*-XGase, as opposed to background contamination. Products were subsequently derivatised with a fluorophore and separated by electrophoresis.

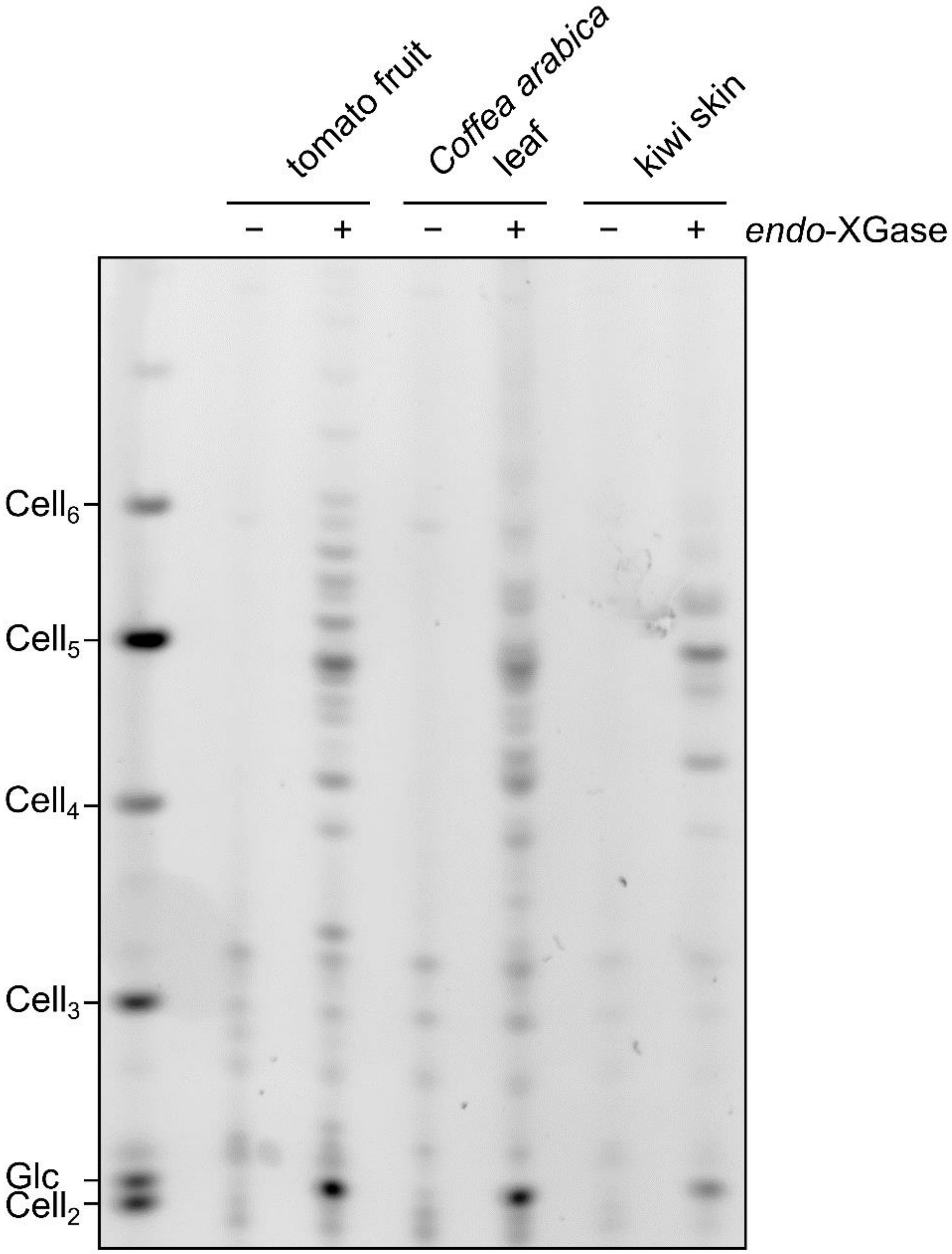

Fig. S17 Sensitivity of *endo*-XGase products from *Catharanthus roseus* leaf xyloglucan to various *exo*-glycosidases. Alkali-extracted hemicellulose from *C. roseus* leaf was digested with *Aa*XEG *endo*-XGase before being subjected to ethanol precipitation. The oligosaccharide products underwent a combinatorial digestion with *Bb*AfcA α1,2-fucosidase (α1,2-Fuc-ase), Fam35 β-galactosidase (β-Gal-ase) and *Bo*GH43A. Products were subsequently derivatised with a fluorophore and separated by electrophoresis.

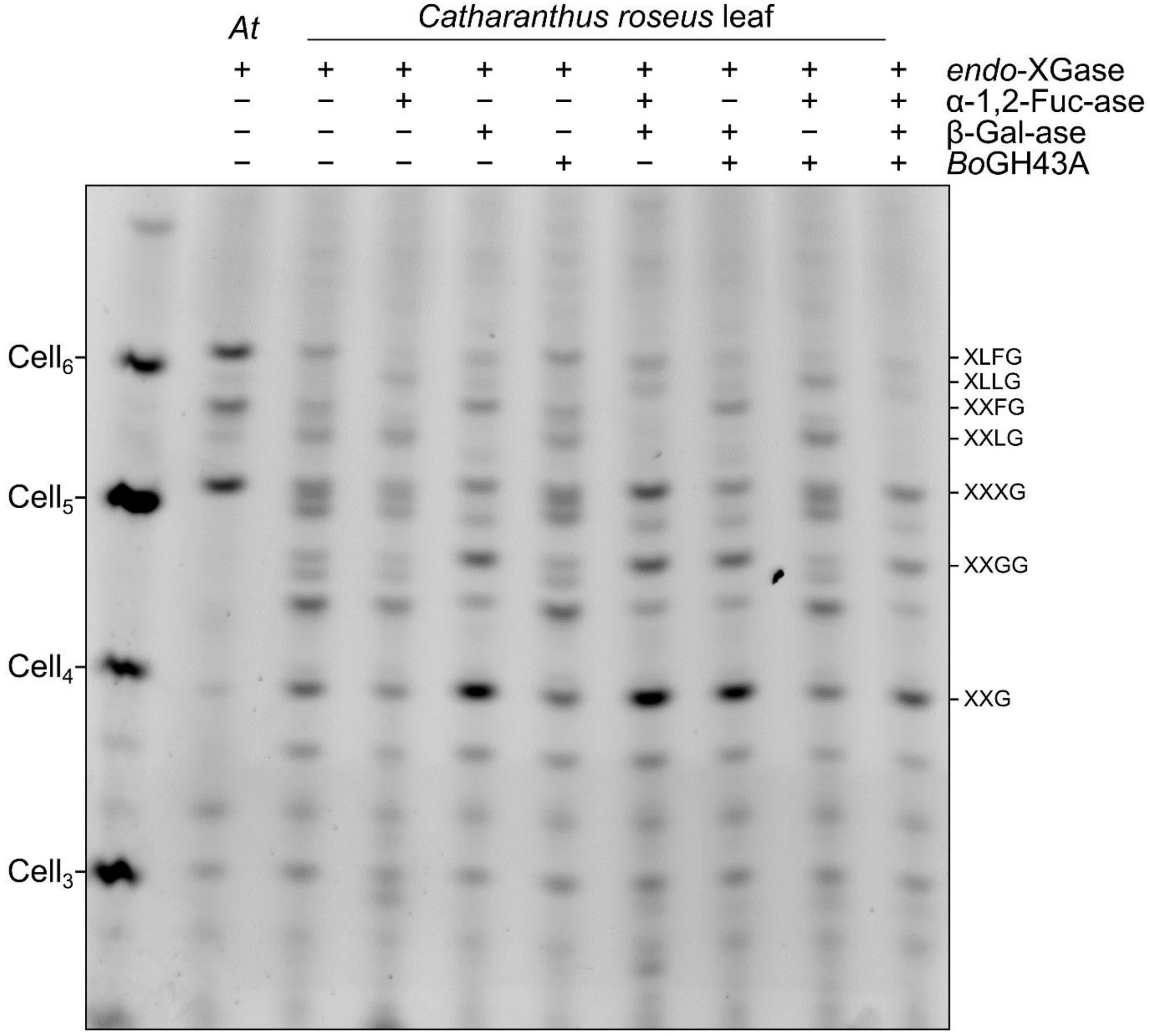

Fig. S18 Sensitivity of *endo*-XGase products from *C. arabica* 'Catimor' root xyloglucan to various *exo*-glycosidases. Alkali-extracted hemicellulose from *C. arabica* 'Catimor' root was digested with *Aa*XEG *endo*-XGase before being subjected to ethanol precipitation. The oligosaccharide products underwent a combinatorial digestion with *Bb*AfcA α1,2-fucosidase (α1,2-Fuc-ase), Fam35 β-galactosidase (β-Gal-ase) and *Bo*GH43A. Products were subsequently derivatised with a fluorophore and separated by electrophoresis.

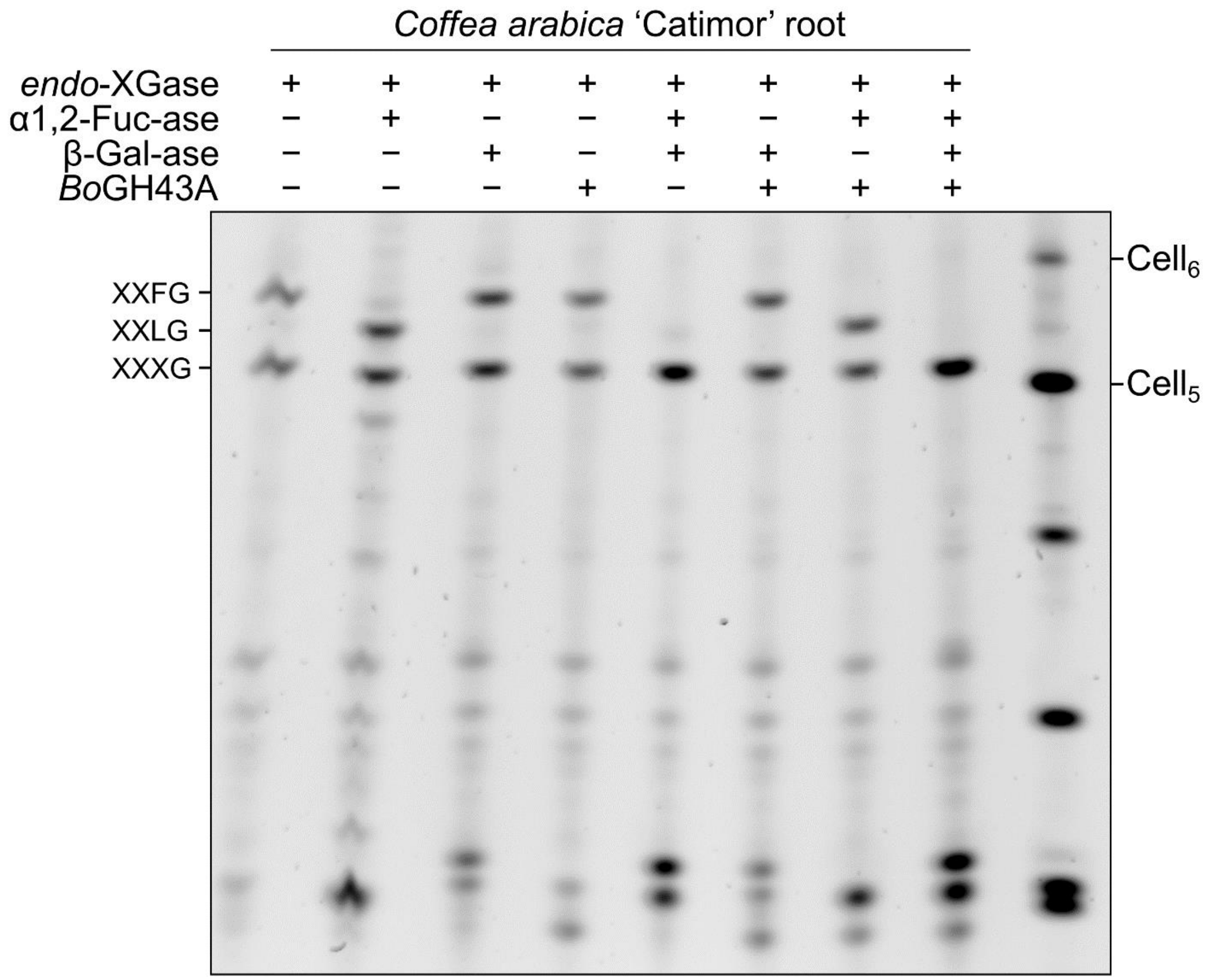

Fig. S19 Sensitivity of *endo*-XGase products from *C. arabica* 'Catuai Amarelo' root xyloglucan to various *exo*-glycosidases. Alkali-extracted hemicellulose from *C. arabica* 'Catuai Amarelo' root was digested with *Aa*XEG *endo*-XGase before being subjected to ethanol precipitation. The oligosaccharide products underwent a combinatorial digestion with *Bb*AfcA α1,2-fucosidase (α1,2-Fuc-ase), Fam35 β-galactosidase (β-Gal-ase) and *Bo*GH43A. Products were subsequently derivatised with a fluorophore and separated by electrophoresis.

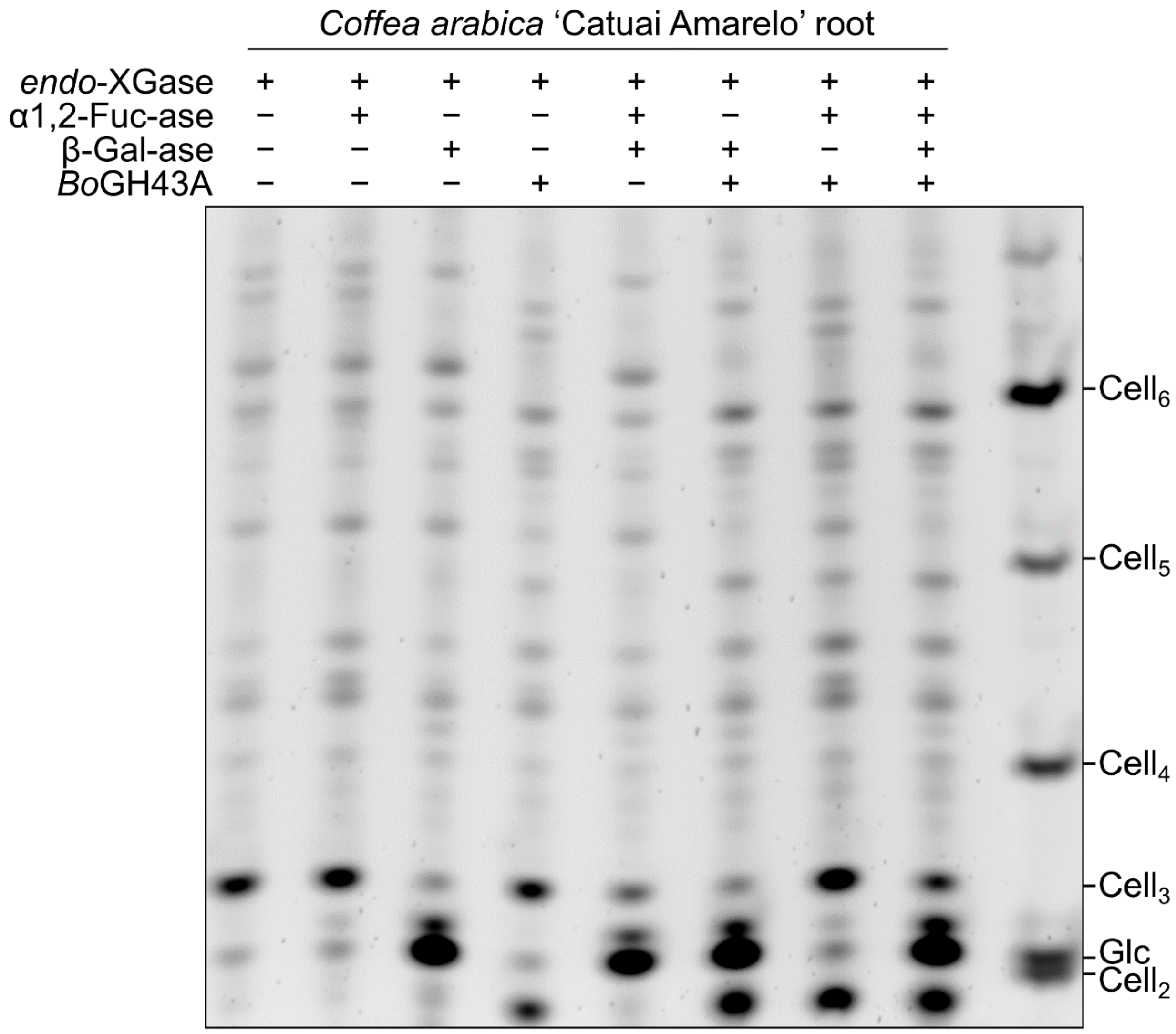

Fig. S20 Sensitivity of *Coffea arabica* 'Catuai Amarelo' root *endo*-XGase products to α1,2/3-arabinofuranosidase and β1,2-xylosidase. Alkali-extracted hemicellulose from *C. arabica* 'Catuai Amarelo' root was digested with *Aa*XEG *endo*-XGase before being subjected to ethanol precipitation. The oligosaccharide products underwent a combinatorial digestion with Fam35 β-galactosidase (β-Gal-ase), *Cj*Abf51 α1,2/3-arabinofuranosidase (α1,2/3-Ara*f*-ase), and/or *Cg*GH3 β1,2-xylosidase (β1,2-Xyl-ase). Products were subsequently derivatised with a fluorophore and separated by electrophoresis.

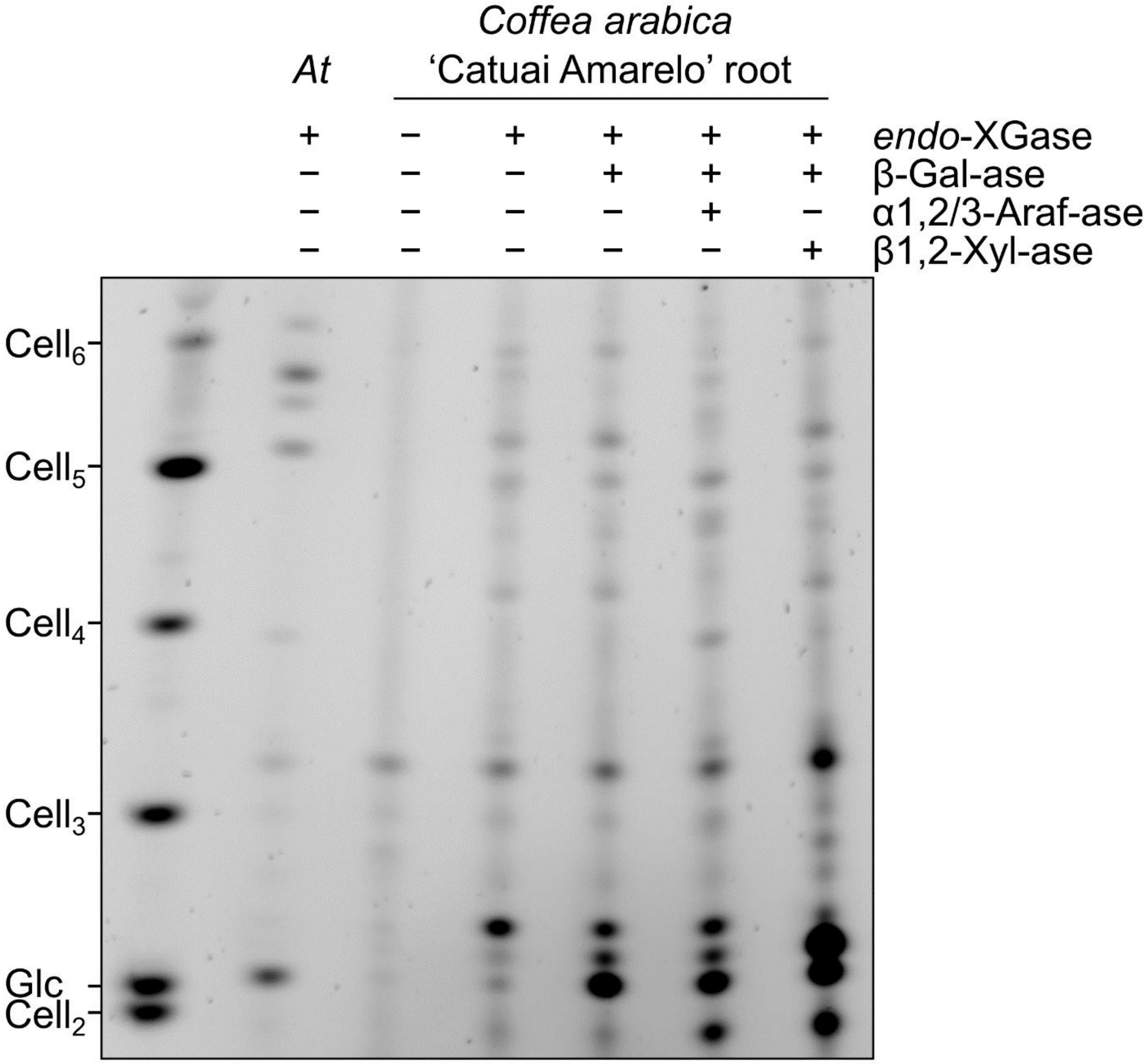

Fig. S21 Characterisation of *endo*-XGase products from kiwi fruit skin xyloglucan. Alkali-extracted hemicellulose from kiwi fruit skin was digested with *Aa*XEG *endo*-XGase before being subjected to ethanol precipitation. **a** PACE gel comparing *endo*-XGase products released from xyloglucan of Arabidopsis, tomato, and kiwi. **b** Combinatorial digest of *C. arabica endo*-XGase products with *Bb*AfcA α1,2-fucosidase (α1,2-Fuc-ase), Fam35 β-galactosidase (β-Gal-ase), and/or *Bo*GH43A.

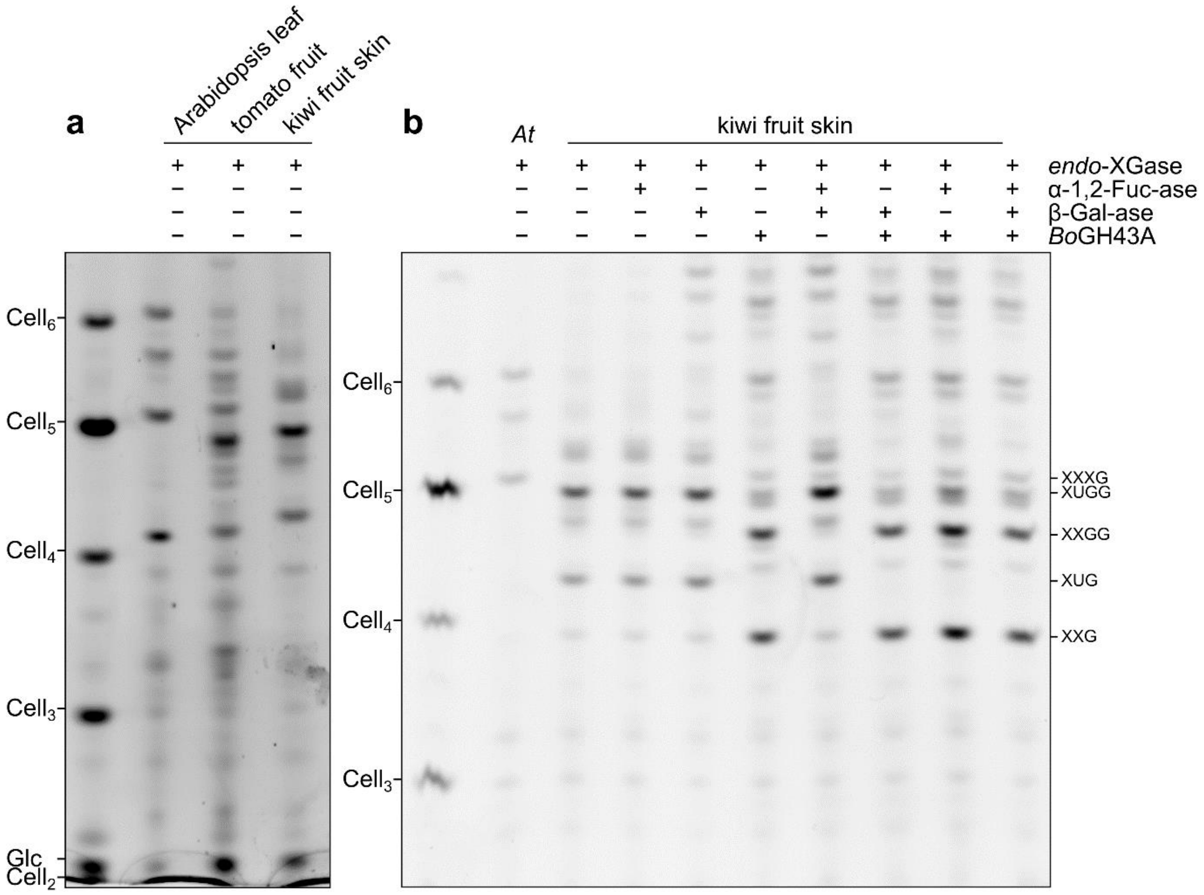

Table S1 Genomic data sources. Predicted protein sequences (primary transcripts only) were downloaded from the listed genomic data sources. For species marked with an asterisk, only nucleotide sequences were available.

(Table provided separately)

Table S2 De novo-synthesised coding sequences for Golden Gate assembly. For VmXST1, codons 167–193 (stricken through) were intentionally omitted from the synthesised construct.

*Vm*XST1

cactctgtggtctcaaATGGCGAATACCTCGTTCCTTCCAACATTTGGCCACCCGTCGACAAATCTCCAAGGCCTAAAAAAGCAGCCGAATAAATCCCCACCCCTTAATT
CCACACTAAAATCATTCCTACACTCCCTTACTCCAACCCGTCGAACGTTAGTCATCATCGTCATCCTTTTCAATGTATTCCTCGTTTTATACCTTACTCGTacccacattc
tctctccatccGAATCCCCTCTAGCATCTCCCGAATTCCTACCCGGAGAATCTAGGATAGTACGAAATATCTCATCGCAAACGTACAGTGGTTCCGGCAGCTTGCAAA
ACAAAGGCGTATTGAATATCTCATCGCAAGCTTACGAGTTACACGACAGCTCGCCGGACTATGACGCACCGGATATCTCATCCCAAACTTATGGCAGATTGCGA
AACAAAGGGGTAGTGGATATCGAATCGCAAAATCACGGCAGCTTGGCGAATAAAGGCGTAGTGAATATCTCATCGGAAAC~~TTACGGCAGCTTGGCGAACAAA~~
~~GAAGTATTGAATATCTCATCGGAAGCGAACAAAGGCATAGTGAATATCTCATCGGAAAG~~TTATGGCAGCTTGGCGAACAAAGAAGTATTGAATATCTCATCGGA
AGCGAACAAAGGCGTAGAGAATATCTCATCGGGACCTTACGGCGGCTTGGCAAACAAAGACGGATTGAATATCTCATCGGAAACTTTCGGTGGCTTGCCGAAC
AAAAGCTGCGATTCCGGTCGGATTTACGTCTATGATCTCCCTCCGATATTGAATTACGATATCAAGAATAATTGCACCCAACTAGAGCCGCCGGACGACAAATGT
GACAAACTTTTGAACGACGGTCTTGGACCGGTGGCGAAAGAGTACGCTGGAATGTTTCCGGAGAGTATTCTCCCTGCGTTCTACTGGACCGATCTTTATTGGGG
CGAGGTGTTGTTTCACAATCGGATGCTTAATCACAGGTGCCGAACACTAGAGCCCGAATCCGCTACGGCGTTCTACATACCTTTTTATGCTGGGCTCGATGTTAG
AAAATACTTGCACAATCACACTGCAAGAGAGCGCGATAGGAATCCTGAGATCCTGACTAAGTGGGTCCAGGAGCAGAAATGGTGGAAGAGATCTAACGGCTCC
GATCACATCATCATGCTTGGAAGGATGACGTGGGATTTCCGACGGTTGGCAGACACTGACGCCGATTGGGGTACCAGATTCCTCAATATGGCCGCGATGCAGA
ATGTCATCCGTGTAACCGTCGAGCGAAACGTGTGGGACAACCTAGAAGTCGCTGTACCCTACCCCACCATATTCCACCCCAGATCAGAATCCGATATCATCCAGT
GGCAGAACTTCCTCAGGAGCCGCCGCCGGAACCACCTCTTCACCTTCGTCGGCGGCGGTCGCTCTAAAATCAAGAACGATTTCCGAGCGGTCCTAAAGAACAAG
TGCCTCAACGAGTCAACCGCGTGCCATCACGTGGACTGCTCCGGGAACAACTGCCTCGACGGGAAAACGGCGGTCATGGCGGCGTTTCTCGACTCCGATTTCTG
TCTCCAGCCGAAGGGGGACGGGTTTACGAGAAGGTCGGTTTTCGATTGCATGTTGGCCGGTTCGGTCCCGGTTTTTTTCTGGAGAGGGACGGCTTACTTGCAAT
ATGAGTTGTACTTCACGCCGAATGAACCGGAGAGTTACTCGGTTTTTATCGACCATAACGACGTGCGCAATGGAACGGATATAAGGAGAGTGTTAGAAGGGTA
TGGGAGAGAGGAAGTGgaaaggatgagagagaaagtaataGAATACATACCGAGATTTGTGTATGCGAAACCGAGTCAAGGGTTGGAGAAAACAAGAGATGCCTTTG
ATATTGCTATTGATGGAGTATTGACCAAATACAAAGCTCACATGGAGAGAGGTAGGGTTGGGAACTGGAATGACATTagttcgtgagaccacgaagtg

*Cc*XBT1 (Cc07_g06550)

cactctgtggtctcaaATGCTTCCCCTTTCCAATTCCTCGTCACAAGTTAAGGACCGCGGTGTCCATGGGAATCCCAAGCGTACTATTACTGCGTTCGATTTCAAGAAG
GCGCTTAATTTTGTCCGCTCCCATGTTTCTTTCCACCAATTCGCATGGGCCCTCATTCTTTTTCTCTTGGAATTTTGTATCTTCCTAGCCTTCACTGCCACCACTCGTG
CTCCCAGTCCTCCCACAGCCGCCGACCGATTGTCGCCCGTCCGTCGTCCCCAGCTCCAGGGGACGCCGCCGGAACAGGTTAGTCAACAGCCGCTGGTTCCTCCCC
ATCATCCCGATGACCGAGCACAATGCAAGTATGGAAGGGTTTATGTATACGACCTTCCTCCAATTTTCAACAAGAAGCTGCTGGAGAATTGCCAGGACTTAGAC
CCCCGGAGATCCCAGTGCGCGGCGGTGTCCAACGACGGGTTCGGACCAAACGCCACTACGCTCGCCGGCACGGTCCCGCGAGAGCTCGCTCATGCTTGGTACT
GGACCGACCTTTTCGCCGGCGAGATTATCTTTCACGCTCGGATCTCCACCCACAGGTGCAGAACCATGGAGCCAGAATCGGCTGCGGCATTCTACGTGCCCTTTT
ACGCTGGGCTGGCGGTTTCCAAGTATCTGTTCACAAACTACACTGCGAAAGAGCGCGATGCTCCGTGCCAGAATTTGCTCCGGTGGATCAAGGGCCAACCGCAT
TGGAAGAGATCCAACGGTTCTGATCATTTTCTTATGCTGGGGCGTAGTTCGTGGGATTTTAGACGGGCCCGCGATGGAGACTGGGGGACCAGCTTCCTCCTCAT
GCCCCTCATGAGACAAATGTTCCGCTTAACGATAGAAAAGAGCCTTGGCGACCCACTGGAAGTCAGCGTGCCCTATCCCTCCGGATTCCATCCCCGGACCACCTC
CGAAGTTGGGCAGTGGCAGGAATTTGTTCGGAGTCGTAAACGGTCGAGCCTCTTCACGTTTGTTGGCGGAAAACGCGGGTATATCAAGAACGATTTCAGGGCT
TTGTTGCTGGATCAGTGTTACGAGGAGTCGGATTCGTGCAAAGCTGTGGACTGTGCCAAGACCCCATGCTTGGACGGCGCTTCCTCGGTCCTGGACGCCTTTCT
GGACTCGGACTTTTGTCTGCAGCCCAGGGGGGACTCGGTCACCAGGAGGTCCACGTTCGACTGCATGCTGGCCGGCTCCATACCCGTTTTTTTCTGGGAGGGAA
CCGTTGGGGGCCAGTACGAGCTGTACATGTCTGATCAAACCGAGTCCTTTTCGGTTTTCATCCACCGGAATAAAGTGAGGAACGGGACTTCGATAAGGAAAGTG
TTGGAGGGATACAGCAGGGAGGACGTCAAGAGGATGAGGGAGAAAGTGATTGACATGATCCCGAGGATTTCGTACGCTTTTCCAGCTGCGGAGGGAGGATTG
GGCAATCTCAAAGATGCCTTCGACATAGGCGTGGAGGAAGTGCTGAGAAGAATAGTCAAAAATGCCAATCCATACAGGTCGGGAGTCGGGACCCGTATCATGA
ATGAGATCACATTCCCAGAGagttcgtgagaccacgaagtg

Cc07_g06570

cactctgtggtctcaaATGAAAATGCACCCCTTTGCCAAATTCTCATTATCGTCGTCTGGCACTAGTAGCAGCAGTAGTAGACGCAATTTTGTGGACAAACCCAAGAAC
CGTCGTGCATGGCTTTTCGTTGCTCTCGTCTTTCCTATTCTCATCCTCCTAATGTTCACCAGTGCTCCGAGACAATATCTATCCATCCGTCGTCTGAAGCTACGTTTC
CGAGCTCCGGCTGGTTCTGAGCAGTGCAAGTACGGCACGGTCTATGTATACGACCTTCCTCCATCCTTCAACAAGAAATTGTTAGACAACTGCCGCGCTATAGAT
CCGCAGAATTCACTTTGCGATGCTCTCTCTAACGATGGGTTTGGCCCGAAAGCCACCGATTTCCAGGGAATCATCCCCGAGGATCTTACTCCCGCGTGGTACGCC
ACTAGCATGTTTGCCGGTGAAGTTATTTATCATACCAGGATCTCCAATTACAAGTGCAGAACCTACGATCCAGATTCTGCAGCAGCTTTTTACATACCCTTTTATG
CGGGATTAGCCACTGCCAAGTATCTGTACGCTAATCGCAGTACTGAGAGCGAGAGAGAATCGCAGGGCGAGAGCTTGGTCAAGTGGGTAACTGAGCAACCGT
CTTGGAAGAGATCCAACGGCGCAGATCACTTTATTATGCTGGGCAGAGTATCTGGGGATTTCAGGCCTGGGGATCATCATATGGATGCAAAATCGGGGTCGAG
CTTCGTTCTCATGCCTCCCATCAGACAGACGCTAAGGCTGGCACTTGAACGAAGCCCCTGGGACCGCTACGAGATTGGGGTGCCGTATCCTACGGGATTTCATCC
CAGGTCCAACGCTGAGCTTGAGAAATGGTTGAAATTCGTGAGAACTCGCAACCGGTCCAAGCTCTTCACATTTGTGGGAGGGAAAGAAAAAAGGTTGAAGGAT
GACTTCAGGGGTTTGTTGGTGGACCAGTGCCGCGACGAGTCTGACAGGTGCACAGTTGTGGACTGTTCGGAGACTCCGTGTTCCGATGGAAGCCCGGCAATTC
TTGAACCCTTCCTGGGCTCCAACTTCTGTTTGCAGCCCAGATCAGGTGATTCTTTCACGAGAAGATCCACATTTAATTGCATGCTGGCTGGTTCAATACCGGTTTT
CTTCTGGAAAAGAAGCATCTTTGGTCAGTATGACTGGTTCCTGAGAGATGACCCCGAGCGGTTCTCGGTATTTATAGACGAGAACCAAGTGAGAAACGGGACA
ATTTCTATTAGGAAAGTGTTGGAGGGATATGGCATTGAAGAGATTCAGATGATGAGGGAAAGGGTCACTAATCTGATACCTAGGTTTCTTTATGTTATGCCCGG
TGATGATGAATACGATGATGGTGGTGTGGGGAAGTATACCATGAGAGATGCAGTTGATATAGCTGTTGAAGGGGTGCTGCGACGATTCAAGGAGCTAAGATT
AGTCGAGCGACAAagttcgtgagaccacgaagtg

Table S3 PCR primers used to amplify DNA parts for Golden Gate assembly.

Target		Sequence (5'→3')
XXT2 promoter		
	Forward	CACTCTGTGGTCTCAGGAGGACTGGTTTGATGTTTTTCATGATATAAACT
	Reverse	CACTTCGTGGTCTCACATTCTTCTTTCTTCTTACAAGATTCTCTGTAAAA
CESA3 promoter		
	Forward	CACTCTGTGGTCTCAGGAGGGTACGAGAGTTACGAAGCAG
	Reverse	CACTTCGTGGTCTCACATTTTGTCACTTAGTTGCTTCCAAC
*Sl*XST1 CDS		
	Forward	GTGGTCTCAAATGTTGCCATCTGAAAATTCTTCCCC
	Reverse	GTGGTCTCACGAACTTAGTTTTTGTTGCTTGAATCTC

Table S4 Exo-glycosidases used in this work.

(Table provided separately)

Table S5 1H and 13C NMR assignments for XUXG oligosaccharide.

Residue		H1/C1	H2/C2	H3/C3	H4/C4	H5/C5	H6/C6
A	α-D-Xyl*p*	5.141/99.08	3.614/81.38	3.870/72.69	3.652/70.26	3.563/3.717/62.07	
B	α-D-Xyl*p*	4.949/99.51	3.542/72.37	3.686/73.96	3.616/70.39	3.571/3.723/62.26	
C	α-D-Xyl*p*	4.946/99.12	3.533/72.28	3.713/73.88	3.607/70.37	3.565/3.732/62.13	
D	β-D-Glc*p*	4.569/101.23	3.45/75.5	3.693/74.97	3.738/79.97	3.818/74.18	3.893/3.976/66.9
E	β-D-Glcp_{nr}	4.56/103.66	3.322/73.82	3.52/76.29	3.539/70.3	3.685/75.01	3.766/3.950/66.68
F	β-D-Glc*p*	4.539/103.14	3.374/74.39	3.666/74.69	3.784/79.98	3.819/74.71	3.908/3.960/66.44
G	β-D-Xyl*p*	4.53/105.64	3.35/74.16	3.448/76.45	3.644/74.91	3.304/3.960/65.91	

Table S6 Kinetic parameters for BoGH43A/B activity on para-nitrophenyl glycosides. Reactions were carried out in 50 mM HEPES, pH 7.5, at 20 °C with 1 µM enzyme. Michaelis-Menten kinetics were collected from initial velocities measured via absorbances at 405 nm. *Parameters extrapolated due to limit of substrate solubility.

Enzyme	Substrate	k_{cat} (s^{-1})		K_M (mM)		k_{cat} / K_M ($M^{-1}s^{-1}$)
*Bo*GH43A	*p*NP-α-Ara*f*	0.01	± 2×10^{-4}	0.49	± 0.04	20

	*p*NP-β-Xyl	0.1	± 4×10^{-4}	8.9	± 0.04	10
*Bo*GH43B	*p*NP-α-Ara*f**	0.01	± 2×10^{-3}	17	± 4	0.8
	*p*NP-β-Xyl*	0.02	± 2×10^{-3}	35	± 2	0.7

Methods S1 Glycosyl hydrolase expression and purification. Plasmids pET-YSBLIC-BoGH43A and pET21a-BoGH43B were transformed into chemically competent E. coli BL21(DE3) (New England Biolabs, Ipswich, MA, USA) according to the manufacturer's protocol and grown on lysogeny broth (LB) agar plates supplemented with 50 mg l−1 kanamycin (BoGH43A) or 100 mg l−1 carbenicillin (BoGH43B) at 37 °C overnight. Colonies were washed off the plate with LB medium and used to inoculate one litre of LB medium (50 mg l−1 kanamycin for BoGH43A or 100 mg l−1 carbenicillin for BoGH43B; baffled flask), which was incubated at 37 °C and 180 rpm until OD600 = 0.7 (BoGH43A) or OD600 = 0.5 (BoGH43B) was reached. Cultures were cooled down, induced with IPTG (0.2 mM for BoGH43A and 0.1 mM for BoGH43B) and shaken (180 rpm) for 18 h at 20 °C (BoGH43A) or 18 °C (BoGH43B). Cultures were harvested by centrifugation at 4000 × g, 15 °C, for 20 min. Pellets were resuspended in Ni-NTA buffer A (BoGH43A: 50 mM HEPES pH 7, 0.3 M NaCl, 10 mM imidazole; BoGH43B: 50 mM HEPES pH 7, 0.5 M NaCl, 30 mM imidazole) supplemented with 2.5 mM MgCl2, Benzonase (5 µl of the solution provided by the supplier; Merck, Darmstadt, Germany) and 1 mg ml−1 lysozyme. Following incubation for 30 min rolling at 4 °C, lysis was completed by sonication (20 cycles, 10 s pulse, 30 s off) on ice. The supernatant was cleared by centrifugation (17,000 × g, 4 °C, 40 min) and loaded onto a Ni-NTA gravity column (column volume, CV: 2 ml) equilibrated in Ni-NTA buffer A. The column was washed with 30 CV Ni-NTA wash buffer (BoGH43A: 50 mM HEPES pH 7, 0.3 M NaCl, 20 mM imidazole; BoGH43B: 50 mM HEPES pH 7, 0.5 M NaCl, 30 mM imidazole) and protein eluted with 3 CV Ni-NTA elution buffer (BoGH43A: 50 mM HEPES pH 7, 0.3 M NaCl, 500 mM imidazole; BoGH43B: 50 mM HEPES pH 7, 0.5 M NaCl, 500 mM imidazole). The protein solution was concentrated in an Amicon centrifugal filter (30 kDa cut-off; Merck), buffer exchanged to size-exclusion (SEC) buffer (BoGH43A: 25 mM HEPES pH 7, 0.1 M NaCl, 1 mM DTT; BoGH43B: 10 mM HEPES pH 7, 0.25 M NaCl) using a PD-10 column (GE) and concentrated in an Amicon centrifugal filter (30 kDa cut-off; Merck) to 1 ml. The protein solution was loaded onto a HiLoad 16/60 Superdex 200 prep grade (GE) equilibrated in SEC buffer and after a 40 ml void volume 1 ml

fractions were collected. Fractions containing BoGH43A or BoGH43B were pooled and concentrated in a 30 kDa Amicon centrifugal filter (Merck) to 15 mg ml−1 (BoGH43A) or 10 mg ml−1 (BoGH43B). For storage, glycerol was added (25% final concentration), protein was frozen in liquid nitrogen and stored at −80 °C.

Methods S2 Oligosaccharide purification. To purify the XUXG oligosaccharide, deacetylated hemicellulose was extracted from rosette leaf AIR of six-week-old CcXBT1-expressing plants (as described above), applying 100 mg AIR to the PD-10 column. To the 3.5 ml eluate, 50 µl 14 µM AaXEG, 20 µl 9.5 µM BbAfcA α1,2-fucosidase, and 20 µl 25 µM Fam35 β-galactosidase were added before incubating at 37 °C for 18 h. The digest was then dried and resuspended in a total of 100 µl 50 mM ammonium acetate, pH 6.0. This sample was applied to a 500 ml Bio-Gel P-2 (BioRad, Hercules, CA, USA) gravity column equilibrated with the same ammonium acetate buffer. Two-millilitre fractions were collected at a flow rate of approximately 0.1 ml min−1. Fractions were analysed by drying a 25 µl aliquot, labelling in 5 µl fluorescent labelling reagent and visualising by PACE (as described above). The XUXG-containing fractions were pooled, further purified using a PD MiniTrap G-10 column (Cytiva, Marlborough, MA, USA) equilibrated to the same buffer, and dried. A similar method was used to enrich the blueberry XyGO of interest, applying 200 mg blueberry skin AIR to each of two PD-10 columns. To each 3.5 ml eluate, 75 µl 14 µM AaXEG was added, and the samples were incubated at 37 °C for 18 h. For precipitation, 6.5 ml of ethanol was added to both aliquots, followed by incubation, centrifugation, and drying of the supernatant as described above. Both samples were then combined in a total volume of 200 µl ammonium acetate buffer; subsequently, 30 µl 9.5 µM BbAfcA, 30 µl 25 µM Fam35 β-galactosidase, and 15 µl 25 µM BoGH43A were added. The reaction was incubated at 37 °C overnight. Ethanol was then added to a final concentration of 70 % before another round of precipitation, centrifugation, and drying of the supernatant. The sample was then resuspended in 1 ml ammonium acetate buffer; size exclusion chromatography then proceeded as above.

References

Beylot M-H, McKie VA, Voragen AGJ, Doeswijk-Voragen CHL, Gilbert HJ. **2001**. The Pseudomonas cellulosa glycoside hydrolase family 51 arabinofuranosidase exhibits wide substrate specificity. *Biochemical Journal* **358**: 607–614.

Denoeud F, Carretero-Paulet L, Dereeper A, Droc G, Guyot R, Pietrella M, Zheng C, Alberti A, Anthony F, Aprea G, *et al.* 2014. The coffee genome provides insight into the convergent evolution of caffeine biosynthesis. *Science* **345**: 1181–1184.

Beylot M-H, McKie VA, Voragen AGJ, Doeswijk-Voragen CHL, Gilbert HJ. **2001**. The Pseudomonas cellulosa glycoside hydrolase family 51 arabinofuranosidase exhibits wide substrate specificity. *Biochemical Journal* **358**: 607–614.

Denoeud F, Carretero-Paulet L, Dereeper A, Droc G, Guyot R, Pietrella M, Zheng C, Alberti A, Anthony F, Aprea G, *et al.* 2014. The coffee genome provides insight into the convergent evolution of caffeine biosynthesis. *Science* **345**: 1181–1184.

Dong A-X, Xin H-B, Li Z-J, Liu H, Sun Y-Q, Nie S, Zhao Z-N, Cui R-F, Zhang R-G, Yun Q-Z, *et al.* 2018. High-quality assembly of the reference genome for scarlet sage, Salvia splendens, an economically important ornamental plant. *GigaScience* **7**: 1–10.

Filiault DL, Ballerini ES, Mandáková T, Aköz G, Derieg NJ, Schmutz J, Jenkins J, Grimwood J, Shu S, Hayes RD, *et al.* 2018. The Aquilegia genome provides insight into adaptive radiation and reveals an extraordinarily polymorphic chromosome with a unique history. *eLife* **7**: e36426.

Iorizzo M, Ellison S, Senalik D, Zeng P, Satapoomin P, Huang J, Bowman M, Iovene M, Sanseverino W, Cavagnaro P, *et al.* 2016. A high-quality carrot genome assembly provides new insights into carotenoid accumulation and asterid genome evolution. *Nature Genetics 2016 48:6* **48**: 657–666.

Katayama T, Sakuma A, Kimura T, Makimura Y, Hiratake J, Sakata K, Yamanoi T, Kumagai H, Yamamoto K. **2004**. Molecular cloning and characterization of Bifidobacterium bifidum 1,2-α-L-fucosidase (AfcA), a novel inverting glycosidase (glycoside hydrolase family 95). *Journal of Bacteriology* **186**: 4885–4893.

Khayi S, Azza NE, Gaboun F, Pirro S, Badad O, Claros MG, Lightfoot DA, Unver T, Chaouni B, Merrouch R, *et al.* 2020. First draft genome assembly of the Argane tree (*Argania spinosa*). *F1000Research 2020 7:1310* **7**: 1310.

Lamesch P, Berardini TZ, Li D, Swarbreck D, Wilks C, Sasidharan R, Muller R, Dreher K, Alexander DL, Garcia-Hernandez M, *et al.* 2012. The Arabidopsis Information Resource (TAIR): improved gene annotation and new tools. *Nucleic Acids Research* **40**: D1202–D1210.

Larsbrink J, Rogers TE, Hemsworth GR, McKee LS, Tauzin AS, Spadiut O, Klinter S, Pudlo NA, Urs K, Koropatkin NM, *et al.* 2014. A discrete genetic locus confers xyloglucan metabolism in select human gut Bacteroidetes. *Nature* **506**: 498–502.

Li M, Zhang D, Gao Q, Luo Y, Zhang H, Ma B, Chen C, Whibley A, Zhang Y, Cao Y, *et al.* 2019. Genome structure and evolution of Antirrhinum majus L. *Nature Plants 2019 5:2* **5**: 174–183.

Okuyama M, Mori H, Chiba S, Kimura A. **2004**. Overexpression and characterization of two unknown proteins, YicI and YihQ, originated from Escherichia coli. *Protein Expression and Purification* **37**: 170–179.

Ouyang S, Zhu W, Hamilton J, Lin H, Campbell M, Childs K, Thibaud-Nissen F, Malek RL, Lee Y, Zheng L, *et al.* **2007**. The TIGR Rice Genome Annotation Resource: improvements and new features. *Nucleic Acids Research* **35**: D883–D887.

Polashock J, Zelzion E, Fajardo D, Zalapa J, Georgi L, Bhattacharya D, Vorsa N. **2014**. The American cranberry: First insights into the whole genome of a species adapted to bog habitat. *BMC Plant Biology* **14**: 165.

Reyes-Chin-Wo S, Wang Z, Yang X, Kozik A, Arikit S, Song C, Xia L, Froenicke L, Lavelle DO, Truco M-J, *et al.* **2017**. Genome assembly with in vitro proximity ligation data and whole-genome triplication in lettuce. *Nature Communications* **8**: 14953.

Sato S, Tabata S, Hirakawa H, Asamizu E, Shirasawa K, Isobe S, Kaneko T, Nakamura Y, Shibata D, Aoki K, *et al.* **2012**. The tomato genome sequence provides insights into fleshy fruit evolution. *Nature* **485**: 635–641.

Schnorr K, Lange L, Lassen SF. **2000**. Fungal Extracellular Fam35 Beta-Galactosidases. World Intellectual Property Organization, WO2001049878A1. URL: https://patentscope.wipo.int/search/en/detail.jsf?docId=WO2001049878.

Tang CY, Li S, Wang YT, Wang X. **2020**. Comparative genome/transcriptome analysis probes Boraginales' phylogenetic position, WGDs in Boraginales, and key enzyme genes in the alkannin/shikonin core pathway. *Molecular Ecology Resources* **20**: 228–241.

Tryfona T, Sorieul M, Feijao C, Stott K, Rubtsov D V., Anders N, Dupree P. **2019**. Development of an oligosaccharide library to characterise the structural variation in glucuronoarabinoxylan in the cell walls of vegetative tissues in grasses. *Biotechnology for Biofuels* **12**: 109.

Tuomivaara ST, Yaoi K, O'Neill MA, York WS. **2015**. Generation and structural validation of a library of diverse xyloglucan-derived oligosaccharides, including an update on xyloglucan nomenclature. *Carbohydrate Research* **402**: 56–66.

Unver T, Wu Z, Sterck L, Turktas M, Lohaus R, Li Z, Yang M, He L, Deng T, Escalante FJ, *et al.* **2017**. Genome of wild olive and the evolution of oil biosynthesis. *Proceedings of the National Academy of Sciences of the United States of America* **114**: E9413–E9422.

Wu H, Ma T, Kang M, Ai F, Zhang J, Dong G, Liu J. **2019**. A high-quality Actinidia chinensis (kiwifruit) genome. *Horticulture Research* **6**: 117.

Xia E, Tong W, Hou Y, An Y, Chen L, Wu Q, Liu Y, Yu J, Li F, Li R, *et al.* **2020**. The Reference Genome of Tea Plant and Resequencing of 81 Diverse Accessions Provide Insights into Its Genome Evolution and Adaptation. *Molecular Plant* **13**: 1013–1026.
